# Characterization of an A3G-Vif_HIV-1_-CRL5-CBFβ Structure Using a Cross-linking Mass Spectrometry Pipeline for Integrative Modeling of Host–Pathogen Complexes

**DOI:** 10.1016/j.mcpro.2021.100132

**Published:** 2021-08-11

**Authors:** Robyn M. Kaake, Ignacia Echeverria, Seung Joong Kim, John Von Dollen, Nicholas M. Chesarino, Yuqing Feng, Clinton Yu, Hai Ta, Linda Chelico, Lan Huang, John Gross, Andrej Sali, Nevan J. Krogan

**Affiliations:** 1 Department of Cellular and Molecular Pharmacology, California Institute for Quantitative Biosciences, University of California, San Francisco, San Francisco, California, USA; 2 Quantitative Biosciences Institute, University of California, San Francisco, San Francisco, California, USA; 3 Gladstone Institute of Data Science and Biotechnology, J. David Gladstone Institutes, San Francisco, California, USA; 4 Department of Bioengineering and Therapeutic Sciences, University of California, San Francisco, San Francisco, California, USA; 5 Divisions of Human Biology and Basic Sciences, Fred Hutchinson Cancer Research Center, Seattle, Washington, USA; 6 Department of Biochemistry, Microbiology, Immunology, University of Saskatchewan, Saskatoon, Saskatchewan, Canada; 7 Department of Physiology & Biophysics, University of California, Irvine, California, USA; 8 Department of Pharmaceutical Chemistry, University of California, San Francisco, San Francisco, California, USA

**Keywords:** architectures of host–pathogen complexes, cross-linking mass spectrometry, integrative structure modeling, A3G, APOBEC3G, CBFβ, core binding factor beta, CTD, C-terminal domain, DSSO, disuccinimidyl sulfoxide, IMP, integrative modeling platform, NTD, N-terminal domain, VCBC, Vif, CBFβ, elongin-B and elongin-C subcomplex, Vif, viral infectivity factor, Vif_con_, consensus Vif, Vif_LAI_, LAI Vif, XL-MS, cross-linking mass spectrometry

## Abstract

Structural analysis of host–pathogen protein complexes remains challenging, largely due to their structural heterogeneity. Here, we describe a pipeline for the structural characterization of these complexes using integrative structure modeling based on chemical cross-links and residue–protein contacts inferred from mutagenesis studies. We used this approach on the HIV-1 Vif protein bound to restriction factor APOBEC3G (A3G), the Cullin-5 E3 ring ligase (CRL5), and the cellular transcription factor Core Binding Factor Beta (CBFβ) to determine the structure of the (A3G-Vif-CRL5-CBFβ) complex. Using the MS-cleavable DSSO cross-linker to obtain a set of 132 cross-links within this reconstituted complex along with the atomic structures of the subunits and mutagenesis data, we computed an integrative structure model of the heptameric A3G-Vif-CRL5-CBFβ complex. The structure, which was validated using a series of tests, reveals that A3G is bound to Vif mostly through its N-terminal domain. Moreover, the model ensemble quantifies the dynamic heterogeneity of the A3G C-terminal domain and Cul5 positions. Finally, the model was used to rationalize previous structural, mutagenesis and functional data not used for modeling, including information related to the A3G-bound and unbound structures as well as mapping functional mutations to the A3G-Vif interface. The experimental and computational approach described here is generally applicable to other challenging host–pathogen protein complexes.

## Highlights

• Integrative modeling using cross-links enables modeling of heterogeneous complexes.
• A pipeline that streamlines modeling of host–pathogen complexes is presented.
• The A3G-Vif_HIV-1_-CRL5-CBFβ integrative structure in solution is determined.
• The structure shows how Vif recruits A3G and the complex structural dynamics.

## Graphical Abstract

**Figure:**
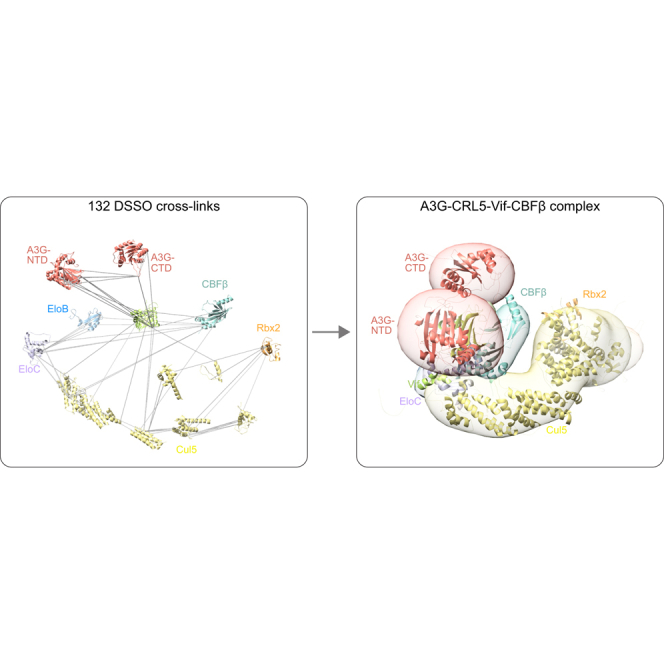

## In Brief

We present a pipeline that streamlines cross-linking mass spectrometry (XL-MS) data collection, data analysis, and integrative modeling of host–pathogen complexes. Using XL-MS, known atomic structures, and functional genetic data, we determined an integrative structure of the HIV-human A3G-CRL5-Vif-CBFβ complex. This structure illustrates HIV-1 Vif interaction with A3G and captures the structural dynamics and flexibility of the entire A3G-CRL5-Vif-CBFβ complex.

Structural heterogeneity plays a crucial role in host–pathogen interactions. Several pathogens exploit the intrinsic disorder of some of their proteins and the structural plasticity of the host proteins to their advantage during infection (1, 2, 3). As a result, host–pathogen protein assemblies are often refractory to traditional structural biology techniques (*i.e.*, X-ray crystallography, NMR spectroscopy, and single particle cryo-electron microscopy). Thus, an alternative approach is needed to solve the structures of these structurally heterogeneous assemblies. Integrative structure modeling, which is based on combining multiple types of input information, is one such approach (4, 5, 6, 7, 8). Here, we have devised a pipeline that streamlines structure characterization of host–pathogen complexes by using integrative structure modeling based on data from cross-linking mass-spectrometry (XL-MS) and residue–protein contacts inferred from mutagenesis studies. We present the application of this pipeline to the structure determination of the A3G-bound Vif-CRL5-CBFβ complex.

XL-MS is a cutting-edge experimental technique that can be used to identify interacting proteins and probe interaction interfaces (9, 10, 11, 12, 13, 14). Chemical cross-linkers that covalently bridge proximal reactive residues are identified using high-resolution MS analysis. Compared with traditional atomic resolution structure determination approaches, XL-MS requires a lower amount of protein, allows for versatile buffer conditions, is relatively robust to protein impurities, and can be applied to compositionally and structurally heterogeneous protein assemblies. One of the major challenges facing XL-MS techniques is the complexity of cross-linked peptide spectra (15), which leads to a large peptide search space for analysis programs. However, the development of MS-cleavable cross-linkers (16, 17, 18) and improved speed and sensitivity of MS^3^ protocols, along with improved computational analyses (14), have made the identification of high-confidence cross-linked peptides more straightforward. Despite these advances, estimating the confidence of each unique cross-link remains challenging. To this end, we developed methods for automatically deconvolving ambiguous spectra assignments as well as methods for quantifying, scoring, filtering, and visualizing cross-linking data, which in turn facilitate use of cross-links in integrative structure modeling.

Integrative structure modeling benefits from multiple types of experimental data to maximize the accuracy, precision, completeness, and efficiency of structure characterization (7, 19, 20). In addition to XL-MS data, the integrative pipeline described here also uses data from mutagenesis studies. For example, mutagenesis studies coupled with two-hybrid interaction mapping techniques (21, 22) can often be used to identify protein–protein interface residues, although they do not provide information about the positions and orientations of the interacting proteins. We developed a restraint on the proximity between residues predicted to be at protein–protein interfaces (PPI), based on functional characterization of mutants.

Members of the APOBEC3 (A3) cytidine deaminase family (*i.e.*, A3D, A3F, A3G, and A3H) can restrict human immunodeficiency virus (HIV) through lethal hypermutation of the HIV genome. The most potent HIV-1 restrictor is A3G (23, 24). Antiviral A3G activity requires that A3G binds to the HIV-1 RNA genome, packages into the virus particle, and has deaminase activity (25, 26, 27). HIV-1 counters this antiviral activity through HIV-1 accessory factor Vif, which binds to A3 proteins preventing their packaging and RNA binding. In addition, Vif recruits the Cullin-5 (Cul5) ring E3 ubiquitin ligase (CRL5) to target A3 proteins for poly-ubiquitylation and degradation by the 26S proteasome (28, 29, 30, 31).

A3G has two domains: an N-terminal domain (NTD) that binds to single-stranded DNA (ssDNA) and RNA (ssRNA) and a catalytic C-terminal domain (CTD) with deaminase activity. While the NTD does not have any catalytic activity, it is required for several antiviral functions, including interaction with Vif (32), A3G dimerization (33, 34), processivity (35), subcellular distribution (36, 37), and packaging into the HIV-1 capsid (38). No atomic structure of the human A3G-Vif complex has yet been determined. Nevertheless, several key residues at the A3G_NTD_-Vif interface have been identified by mutational, functional, evolutionary, and computational studies of various Vif and species homologs or mutant A3Gs (21, 39, 40, 41, 42, 43). However, these studies sometimes appear in conflict with each other; for example, studies have been inconclusive if A3G region 31 to 45 is necessary for binding the full-length and CTD of Vif (21, 44).

While the mechanism for Vif neutralization of A3 family members has been broadly characterized (26, 45, 46), the purification and structural characterization of Vif-CRL5 complexes has been hindered by their instability. Our previous studies that systematically mapped physical interactions between HIV and host proteins using affinity tag purification mass spectrometry (AP-MS) (47) revealed that Vif stably interacts with the cellular transcription factor core-binding factor β (CBFβ). Follow-up studies demonstrated that the CBFβ-Vif heterodimer binds to Elongin B/C complexes to act as the substrate receptor for the CRL5- E3 ligase and that CBFβ is required to stabilize the complex (30). Following this discovery, the structure of the stable Vif-CBFβ-EloB-EloC-Cul5_NTD_ subcomplex was determined by X-ray crystallography (48) along with partial structures of the A3G_NTD_, A3G_CTD_, and Cul5 (supplemental Fig. S1). However, the structure of the full A3G substrate-bound complex, which consists of a full-length Cul5 bound to the E2 docking protein Rbx2, and the Vif-CBFβ-EloB/C (VCBC) adaptor complex, has been refractory to traditional structure determination techniques, likely because of its compositional and/or structural heterogeneity. Additional challenges that may have prevented the structural characterization of the A3G-Vif-CRL5-CBFβ complex include the low stability and solubility of the full-length A3G protein purified from *Escherichia coli* and the intrinsically transient nature of substrate–enzyme interactions.

In this work, we use the lysine-reactive MS-cleavable DSSO cross-linker (17) to obtain a comprehensive set of direct PPIs in the A3G-Vif-CRL5-CBFβ complex. Based on the cross-links, atomic structures of the components (supplemental Fig. S1), and previously published mutagenesis data, we then computed an integrative structure of the A3G-Vif-CRL5-CBFβ complex in solution. The structure was cross-validated based on the data used for modeling and previously published structural, biochemical, and functional data not used for modeling. This study has demonstrated the utility of DSSO-based XL-MS for mapping proximal lysine residues in structurally heterogeneous complexes, in turn enabling integrative structure determination of such complexes. The combined experimental and computational approach described here is generally applicable to other challenging host–pathogen protein complexes.

## Experimental Procedures

### DSSO Cross-linking and Protein Digest

The complexes used in this study that contain either consensus Vif (Vif_con_) or LAI Vif (Vif_LAI_) (*i.e.*, A3G-Vif_con_-CRL5-CBFβ, Vif_con_-CBC-Cul5_NTD_, Vif_con_-CRL5-CBFβ, and A3G-Vif_LAI_CBC) were purified as previously described in (49, 50, 51, 52). Reconstituted complexes were diluted to ~5 μM (different preparations at 1–1.2 mg/ml) in 20 mM HEPES pH 7.5, 300 mM NaCl, 10% Glycerol. Samples were reacted with increasing molar ratios of DSSO (supplemental Table S1) and cross-linking reactions carried out at 37 °C for 30 min at 1000 RPM on an Eppendorf Thermomixer C. All reactions were quenched with 100 mM NH_4_HCO_3_ or 100 mM Tris pH 8.0, then mixed with SDS-PAGE loading dye. Cross-linked samples were analyzed by SDS-PAGE and stained with MS safe blue stain (AcquaStain, Bulldog Bio). Bands corresponding to cross-linked proteins (as compared with non-cross-linked control samples) were excised and subjected to in gel digest with either trypsin or chymotrypsin (supplemental Fig. S2). Cross-linked peptides were analyzed by LC-MS^3^ and identified through database searching as described below.

### Analysis of Cross-linked Peptides by LC-MS^3^

Dried peptide samples were dissolved in 2% FA, 3% ACN, and submitted for specialized LC-MS^3^ analysis using an Easy-nLC 1000 (Thermo Fisher Scientific) coupled to an Orbitrap Elite Hybrid Mass Spectrometer with ETD (Thermo Fisher Scientific). Online peptide separation was performed with a 75 μm × 30 cm fused silica IntregraFrit capillary column (New Objective) packed in-house with 1.9-μm Reprosil-Pur C18 AQ reverse-phase resin (Dr Maisch-GmbH). Peptides were eluted at a flow rate of 300 nl/min using the following gradient of 5% B for 1 min, 5% to 35% B in 50 min, 35% to 95% B in 5 min, and 95% B for 4 min (mobile phase buffer A: 100% H_2_O/0.1% FA; mobile phase buffer B: 100% ACN/0.1% FA). Each individually collected in-gel digest band sample was run in technical duplicate by two similar data-dependent acquisition methods for MS^3^ analysis of cross-linked peptides (based on methods in (16)). For each method, a single acquisition cycle consisted of either 9 or 11 scan events as follows: 1) one full MS^1^ scan in the orbitrap (350–1500 m/z, 120,0000 resolution, AGC target of 1 × 10^6^, max injection time of 100 ms); 2) two data-dependent MS^2^ scans in the orbitrap (15,000 resolution, AGC target of 5 × 10^4^, max injection time of 500 ms) with normalized collision energy set at 22% on the top two precursor ions; and 3) either three or four MS^3^ scans in the ion trap (ion count target 10^4^, max injection time of 50 ms) with normalized collision energy set at 35% on the top three or four ions from each MS^2^ scan. Precursors with charge state 4 and above were sampled for MS^2^ and dynamically excluded for 20 s (tolerance of 10 ppm), with charge state and dynamic exclusion turned off for MS^3^.

### Identification of Cross-linked Peptides

Cross-link peptide identification was carried out in a semiautomated three-step process (supplemental Fig. S3). Briefly, MS^2^ and MS^3^ data were separately extracted from raw files using MSConvert (ProteoWizard) (53, 54). MS^3^ data were searched on a locally installed version of ProteinProspector (v. 5.19.1) for peptide identification and DSSO-remnant modification localization. Exact peptide search and filtering criteria, including scores and E-values, for all trypsin and chymotrypsin data can be found in the *ProteinProspector Batch-Tag* and *searchCompare* setup files, which are provided as xml or txt supplemental Files (bt_trypsin.txt, bt_chymotrypsin.txt, bt_trypsin.xml, bt_chymotrypsin.xml, sc.xml, and sc.txt). MS^2^ and MS^3^ data and peptide identifications are integrated and cross-linked peptides (*e.g.*, dead-end or mono-linked, loop-linked, and interlinked) were identified by XLTools, a revised version of XL-Discoverer (55, 56). Cross-linked peptide data were then summarized with ambiguous assignments deconvoluted and unique and redundant peptides quantified and scored using in house scripts (available at https://github.com/integrativemodeling/A3G-CRL5-Vif-CBFb). For detailed methods and calculations, see Supplementary Information. All of the proteomics datasets from each step, including raw files, MS^2^ and MS^3^ peak files, and MS^3^ search files, have been deposited to the ProteomeXchange Consortium *via* the PRIDE (57) partner repository with the dataset identifier PXD025391. Annotated spectra for all interlinked, loop-linked, dead-end, and single peptides can be found on the MSViewer application through ProteinProspector (58) (https://msviewer.ucsf.edu/prospector/cgi-bin/msform.cgi?form=msviewer) with the search key 9tjmaqhszr.

### Integrative Structure Modeling

We applied an integrative structural modeling approach to characterize the structure of the A3G-Vif-CRL5-CBFβ complex in solution, based on the atomic structures of the components, 132 DSSO cross-links, and previously published mutagenesis data. Integrative structure determination proceeded through the standard four stages (19, 20, 59): 1) gathering data, 2) representing subunits and translating data into spatial restraints, 3) sampling of configurations to produce an ensemble of structures that satisfies the restraints, and 4) analyzing and validating the ensemble structures and data. The integrative structure modeling protocol (*i.e.*, stages 2, 3, and 4) was scripted using the Python Modeling Interface (PMI) package, a library for modeling macromolecular complexes based on our open-source Integrative Modeling Platform (IMP) package (19). Detailed methods can be found in Supplementary Information.

### Validation by Randomly Removing Data and Resampling

The resulting ensemble of models was validated by recomputing it with subsets of the data (*i.e.*, “jackknifing”) (60). We performed sampling runs without the cross-links or mutagenesis data and with only random fractions of the cross-links.

### Data Visualization

The sequence coverage and distributed count of all dead-end, loop-linked, and interlinked (intra- and intersubunit) cross-linked peptide data were visualized using a newly developed tool, XLs Summary Viewer implemented using the Matplotlib Python Package (61) and available in https://github.com/integrativemodeling/A3G-CRL5-Vif-CBFb. All protein structures and integrative models were visualized using Chimera (https://www.rbvi.ucsf.edu/chimera/) (62). MS^3^ spectral data were visualized using the interactive Peptide Spectral Annotator (IPSA-http://www.interactivepeptidespectralannotator.com/BulkDataUpload.html) (63). Interlinked residue data were visualized as a circos linkage map using CX-Circos (v 0.11.1, http://www.cx-circos.net/), from data files automatically generated during step 3 of the cross-link peptide identification pipeline (supplemental Table S2).

### Experimental Design and Statistical Rationale

**Fig. 1:**
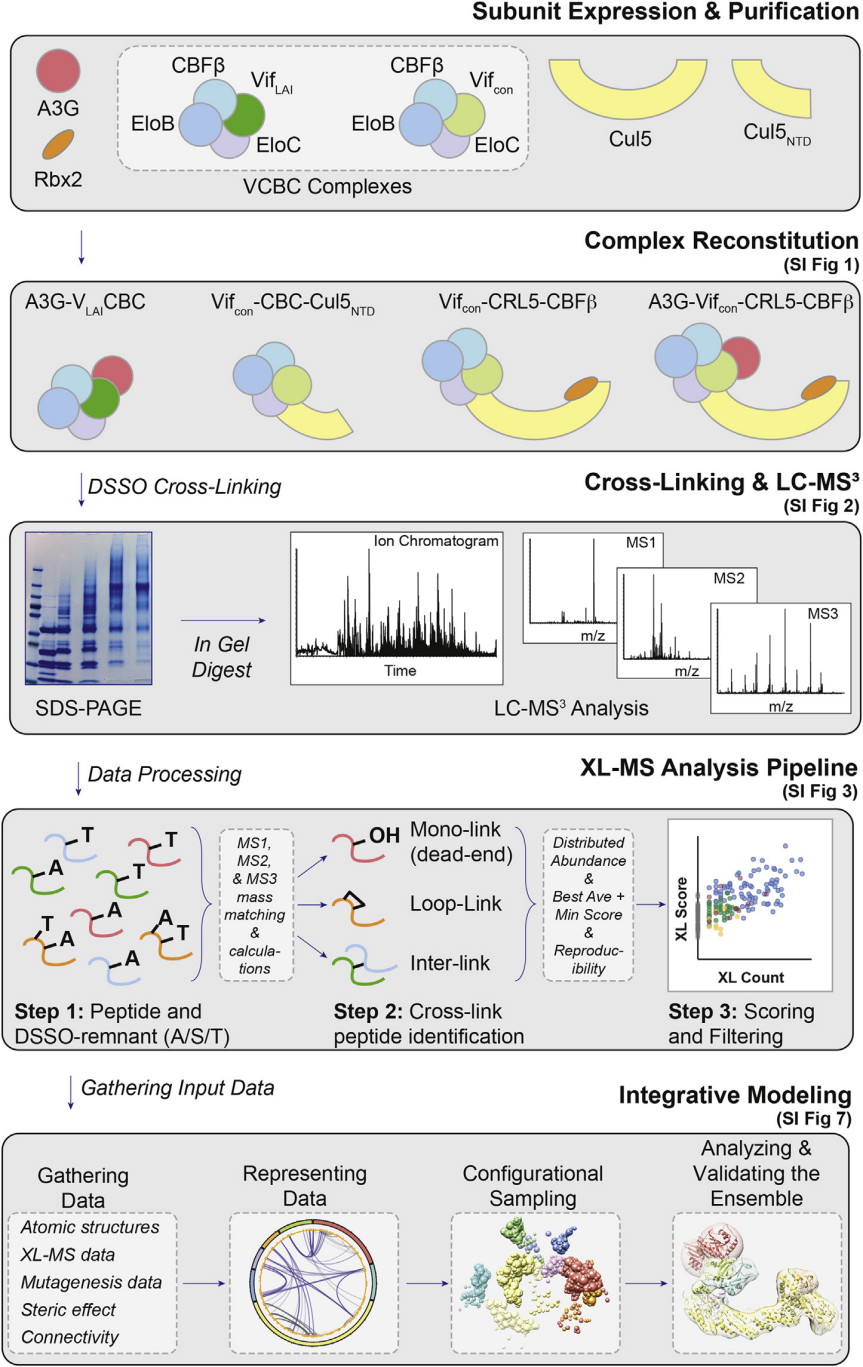
**Overview of the pipeline for integrative modeling of structurally heterogeneous complexes based on cross-linking mass spectrometry (XL-MS).** First, all protein subunits are expressed or coexpressed and purified. Second, the full protein complex and selected stable subcomplexes are reconstituted *in vitro*. For more details on the subunit components and functions, see supplemental Fig. S1. Third, each protein complex is cross-linked using MS-cleavable DSSO cross-linker. Cross-linked protein complexes are then separated by SDS-PAGE and stained gel pieces are excised for in-gel enzymatic digestion (supplemental Fig. S2). The resulting peptide mixtures are separated and analyzed *via* liquid chromatography−tandem mass spectrometric (LC-MS) analysis. Fourth, database searching of the MS data identifies DSSO-modified peptides and their linkage sites. Identified interlinked, loop-linked, and mono-linked (dead-end) modified peptides are quantified, scored, and filtered by our XL-MS analysis protocol (supplemental Fig. S3). Lastly, the cross-link dataset and other available data are used for integrative modeling (supplemental Fig. S7).

Four HIV-1 Vif-containing complexes were expressed, purified, and reconstituted for *in vitro* cross-linking with disuccinimidyl sulfoxide (DSSO, (17)) (Fig. 1). These four complexes include the full-length A3G-Vif_con_-CRL5-CBFβ complex (*i.e.*, A3G, Vif_con_, CBFβ, EloB, EloC, Cul5, and Rbx2), and Vif-containing subcomplexes Vif_con_-CRL5-CBFβ, Vif_con_-CBC-Cul5_NTD_, and A3G-V_LAI_CBC (the number of replicates for each complex and the full experimental details are provided in supplemental Table S1). Each sample was prepared using multiple cross-linking concentrations to obtain a comprehensive set of cross-linked peptides. Each sample cross-linked at different concentrations was separated by SDS-PAGE and cross-linked bands excised for in-gel digest. Samples were digested by trypsin or chymotrypsin in gel, and the digest samples were analyzed by two similar LC-MS^3^ methods to identify dead-end (mono-linked), loop-linked, and interlinked peptides (supplemental Table S1). The full-length A3G-Vif_con_-CRL5-CBFβ complex was prepped five times, three times for trypsin digest (1-A to 3-A in supplemental Table S1) and twice for chymotrypsin digest (1-B and 2-B in supplemental Table S1) to extend cross-linked peptide coverage of the individual components in the complex. The Vif_con_-CRL5-CBFβ and Vif_con_-CBC-Cul5_NTD_ complexes were prepared three and two times for trypsin digest, respectively. The A3G-V_LAI_CBC was prepared only once for trypsin digest. The integrative structures were validated using a series of tests to determine precision and how well they agree with the input information used and not used for modeling (64, 65).

## Results

### Pipeline for Integrative Modeling of Structurally Heterogeneous Host–Pathogen Complexes Based on XL-MS

To characterize the architecture of host–pathogen complexes, we present a pipeline that streamlines integrative structure modeling based on chemical cross-link data and predicted residue–protein contacts from mutagenesis studies (Fig. 1). The general workflow includes expression, purification, and reconstitution of protein complexes, and XL-MS, and integrative structure modeling. To improve the sequence coverage of each subunit and the number of cross-links detected by MS, we included *in vitro* stable subcomplexes in addition to full-length protein holo-complexes. Each reconstituted protein complex is cross-linked using the MS-cleavable, amine reactive chemical cross-linker DSSO (17) that yields a complex mixture of: 1) unmodified peptides; 2) dead-end (or mono-linked) peptides; 3) loop-linked peptides, where two residues on the same intact peptide are linked; and 4) interlinked peptides, where two residues on two individual peptides (referred to as α and β) are linked (supplemental Fig. S4, *A* and *B*). Using specialized multistage liquid chromatography–mass spectrometry (LC-MS^n^) methods, these four types of peptides are identified and measured (see supplemental Fig. S4, *C*–*F* for representative interlink peptide).

Cross-linked peptides that are identified more frequently (*i.e.*, they have a higher redundant count) and where both peptides (*i.e.*, α and β) individually have high scores (*i.e.*, the average and minimum of these two scores are both high) (SI Methods, (66, 67)), represent higher confidence linkages than those with low count and low scores. To quantify this confidence for use in integrative modeling, we define a composite score based on the frequency with which the linkage was identified (redundant count) as well as the highest average and minimum scores for the best representative peptides for each unique linkage. For ambiguous linkages (*i.e.*, cross-linked peptides with missed cleavages and sequentially close lysine residues), the counts were uniquely distributed based on the XL-remnant assignments and proportionally added to the unique residue–residue linkage events count (SI Methods). This deconvolution is inspired by the definition of the distributed normalized abundance factor (dNSAF) (68) that similarly distributed ambiguously assigned peptide spectral counts (based on homologous sequences within a database).

In addition to the cross-links data, we explored how integrative structure determination can benefit from spatial restraints derived from mutagenesis data indicative of interface residues. To this end, we converted the mutagenesis data into an upper bound on the distance between the residues identified to be required for binding and the closest residues in the predicted bound protein (SI Methods). We named this spatial restraint the *residue–protein proximity restraint*. An advantage of this approach is that it does not make assumptions about the orientation of the proteins or the binding interface on the predicted bound protein. Similar restraints have been implemented by other interactive modeling software to incorporate data from mutagenesis into docking protocols (69, 70).

### XL-MS Analysis of A3G-Vif-CRL5-CBFβ

**Fig. 2:**
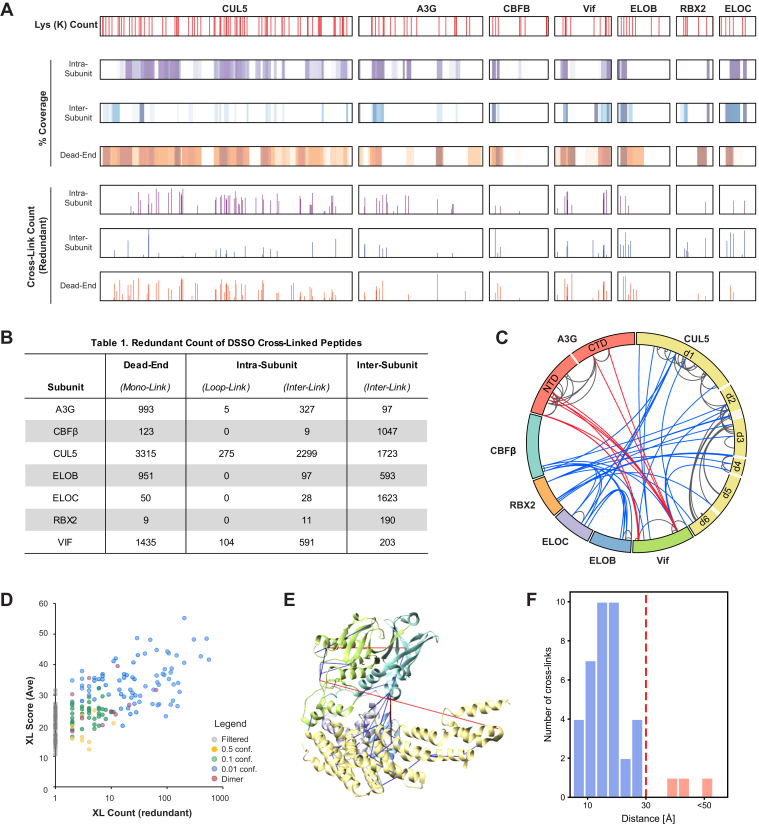
**DSSO XL-MS analysis of the A3G-Vif-CRL5-CBFβ complex and subcomplexes.***A*, overview of cross-linked peptide coverage for all seven proteins in A3G-Vif-CRL5-CBFβ. The position of each lysine (Lys Pos) is shown on the *top panel* with *red lines*. The sequence coverage (% Cov) and redundant cross-link count (XL Count) of each protein are shown below. Residue positions in intrasubunit (*purple*), intersubunit (*blue*), and dead-end (*orange*) linkages are also shown below. *B*, table summarizing the number of redundant cross-links identified for each subunit. *C*, CX-Circos cross-links map of all A3G-Vif-CRL5-CBFβ unique cross-links. Intra- and intersubunit cross-links are represented by *gray* and *blue edges*, respectively. A3G to Vif cross-links are colored *red*. The A3G NTD and CTD rigid bodies used for integrative modeling are labeled on A3G as *darker pink* with the flexible linker being *lighter pink*. The Cul5 domains used as rigid bodies in the dynamic model are labeled on Cul5 as *darker yellow* and the flexible linkers are in *light yellow*. *D*, scatter plot showing the relation between redundant count and the cross-link average alpha and beta peptides identification scores. A high (*blue*), medium (*green*), or low (*orange*) composite confidence score was assigned to each cross-link. Cross-links identified only once (redundant count = 1) or cross-links corresponding to homodimers were filtered out (*gray*). The composite confidence scores were used for integrative structure modeling. *E*, satisfied (*blue*) and violated (*red*) cross-links are mapped onto the X-ray structure of the Vif-CBC-CUL5_NTD_ subcomplex (PDB code 4N9F), using a 30 Å Cα-Cα distance cutoff. *F*, histogram of the distances between cross-linked residues in the X-ray structure. The *vertical dashed red line* represents the 30 Å cutoff used to define satisfied XLs (Cα-Cα distance < 30 Å).

The full-length A3G-Vif_con_-CRL5-CBFβ complex (*i.e.*, A3G, Vif_con_, CBFβ, EloB, EloC, Cul5, and Rbx2), as well as three different Vif-containing subcomplexes (*i.e.*, Vif_con_-CRL5-CBFβ, Vif_con_-CBC-Cul5_NTD_, and A3G-V_LAI_CBC), were cross-linked with DSSO, digested, and analyzed by XL-MS^3^ for peptide and cross-link identification. The subcomplexes allowed us to compare contributions of each protein, look at two different Vif protein variants (*i.e.*, LAI and consensus), and helped to increase our confidence in A3G and Vif-CBC cross-links. We obtained a good overlap of detected cross-links between the A3G-V_LAI_CBC and A3G-Vif_con_-CBC subcomplexes (supplemental Fig. S5). Consequently, the data of all subcomplexes were combined. Reproducible (*i.e.*, uniquely identified in at least in two separate MS runs either from a biological or technical replicate) peptides were included for further cross-links processing and integrative modeling. In total, we identified 6959 dead-end, 6300 interlinked, and 389 loop-linked redundant (nonunique) peptides from all seven components of the A3G-Vif-CRL5-CBFβ complex (supplemental Tables S2–S6). The estimation of the false discovery rate (FDR) was 2.1%, as determined by the percentage of cross-linked peptides for which at least one partner is from the decoy database (1.4%) or from a non-A3G-Vif-CRL5-CBFβ proteins (0.7%). Interlinked peptides had a slightly higher FDR (3%) as compared with dead-end (1%) or loop-linked (1.3%) peptides. Through pooling the data collected from A3G-Vif-CRL5-CBFβ and the other three Vif-containing subcomplexes, we achieved 72% coverage of the protein sequences and 83% coverage of the reactive residues in the full A3G-Vif-CRL5-CBFβ complex (Fig. 2*A*). This coverage includes dead-end modifications and interlinks identified for all seven proteins, indicating good coverage for each individual subunit within the complex. Different subunits demonstrate different proportions of dead-end and intrasubunit or intersubunit linkages (Fig. 2*B*). For instance, A3G shows a higher proportion of dead-end and intrasubunit linkages than intersubunit linkages, indicating that A3G might be involved in less stable PPI interfaces as compared with CBFβ, EloC, or Rbx2, which demonstrate higher percentages of intersubunit linkages (Fig. 2*B*).

Based on the identified cross-linked peptides (supplemental Fig. S5), unique residue-to-residue linkages were determined. To ensure the validity of subsequent analyses, we use high scoring and reproducible residue-to-residue linkages that are observed across biological replicate experiments (SI Methods). A total of 132 reproducible and high confidence cross-links for A3G-Vif-CRL5-CBFβ were classified into three confidence categories and used for subsequent analysis and modeling (supplemental Table S2 and Fig. 2, *C* and *D*).

Of the 132 high-confidence A3G-Vif-CRL5-CBFβ cross-links identified, only 30% (40) could be mapped to the X-ray structure of the Vif-CBC-Cul5_NTD_ subcomplex, with the remaining cross-links mapping to either missing segments or missing subunits (Fig. 2*E*). This mapping revealed that 37 cross-links (93%) are satisfied by the X-ray structure, where satisfied cross-links are defined as having a distance between the Cα of the cross-linked residues less than 30 Å (Fig. 2*F*) (71). This high cross-link satisfaction suggests that the X-ray crystal structure of the Vif-CBC-Cul5_NTD_ subcomplex is similar to the full-length A3G-Vif-CRL5-CBFβ structure in solution. Furthermore, we observed a large number of cross-links between regions known to be at or near the binding interface between Vif and Cul5 and CBFβ and Cul5 (supplemental Fig. S6). The cross-links that are violated may be explained by the conformational heterogeneity of the A3G-Vif-CRL5-CBFβ complex, differences between solution and crystallographic structures, or uncertainty in mass spectrometry identifications. Similarly, we mapped the cross-links to the structure of the Vif-CBFβ-Cul5-Rbx2-EloB-EloC subcomplex (Vif-CRL5-CBFβ subcomplex) for which the Cul5 CTD and Rbx2 subunit were modeled using comparative modeling (72, 73) (SI Results). In this case, 87 (66%) of the 132 unique cross-links mapped to the Vif-CRL5-CBFβ modeled subcomplex and 80% of the cross-links are satisfied (supplemental Fig. S7). The unsatisfied cross-links mostly span residues between the Cul5 NTD and Vif-CBC complex or between Cul5 and Rbx2, indicating that the comparative model of the full-length Cul5 does not fully capture its solution structure or its structural heterogeneity. In addition, 34% of the identified cross-links cannot be mapped to either the Vif-CBC-Cul5_NTD_ X-ray structure or the Vif-CRL5-CBFβ comparative structure, due to segments or components missing from the structure. Thus, we applied integrative structure modeling to determine the full structure and structural dynamics of the A3G-Vif-CRL5-CBFβ complex.

### The Integrative Structure of A3G-Vif-CRL5-CBFβ

To determine the A3G-Vif-CRL5-CBFβ solution structure, we performed integrative structure modeling using the previously described four-stage workflow (SI Methods and supplemental Fig. S8) (7, 19, 20, 74). The input information includes our scored and filtered 132 DSSO cross-links, the X-ray structure of the Vif-CBC-Cul5_N__TD_ subcomplex (48), comparative models of the A3G NTD and CTD, and full-length Cul5 and Rbx2 (SI Methods), and predicted residue–protein contacts from mutagenesis studies (21, 22, 41, 75).

Preliminary integrative modeling represented the Vif-CRL5-CBFβ subcomplex by a single rigid body defined by atomic structure of the Vif-CBC-Cul5_NTD_ subcomplex (48) and comparative models of the full-length Cul5 and Rbx2. Each domain of A3G was represented as a separate rigid body. The linker between the A3G domains (residues 195–200) and regions missing in the X-ray structure were represented by flexible strings of beads (*i.e.*, one residue-per-bead). This representation and output models are hereafter referred to as the rigid representation and model. Models obtained using this representation did not satisfy the spatial restraints implied by the data within the uncertainty of the data (below, SI Methods, supplemental Table S7). Therefore, to characterize the flexibility of the complex, we performed integrative structure modeling of the A3G-Vif-CRL5-CBFβ complex with a modified representation that allowed for relaxation in the configuration of the Vif-CBC subcomplex subunits and for alternative conformations of Cul5 and A3G subunits. Specifically, we represented each protein of the Vif-CBC subcomplex and Rbx2 as independent rigid bodies. Cul5 was represented as six rigid bodies connected by flexible linkers (4–23 residues each) representing the loop between the: (i) second and third repeat of the NTD, (ii) elongated NTD and globular CTD, (iii) 4-helix bundle, (iv) α/β, (v) WH-A, and (vi) WH-B domains (SI Methods, supplemental Fig. S9*A*) (76). The A3G NTD and CTD remain represented as rigid bodies (supplemental Table S8). This representation and output models are hereafter referred to as the flexible representation and model.

With these representations in hand, we next translated the input information into spatial restraints as follows. First, the 132 DSSO cross-links were used to construct a Bayesian term that restrained the distances spanned by the cross-linked residues (SI Methods). Second, we converted the mutagenesis data into residue–protein proximity restraints. Two such restraints were defined corresponding to well-established genetically confirmed regions required for A3G binding and degradation, namely A3G residues 126 to 132 and Vif residues 40 to 45 (21, 22, 41, 75). Third, to use the crystal structure of the Vif-CBC-Cul5_N__TD_ subcomplex and comparative model of Cul5/Rbx2 as templates, we imposed *structural equivalence distance restraints* designed to restrain the model to resemble the templates as much as possible (77). These restraints were applied between pairs of residues closer than 7.0 Å across an interface between two rigid bodies only in the flexible representation. Lastly, we applied sequence connectivity and excluded volume restraints to all components (SI Methods).

**Fig. 3:**
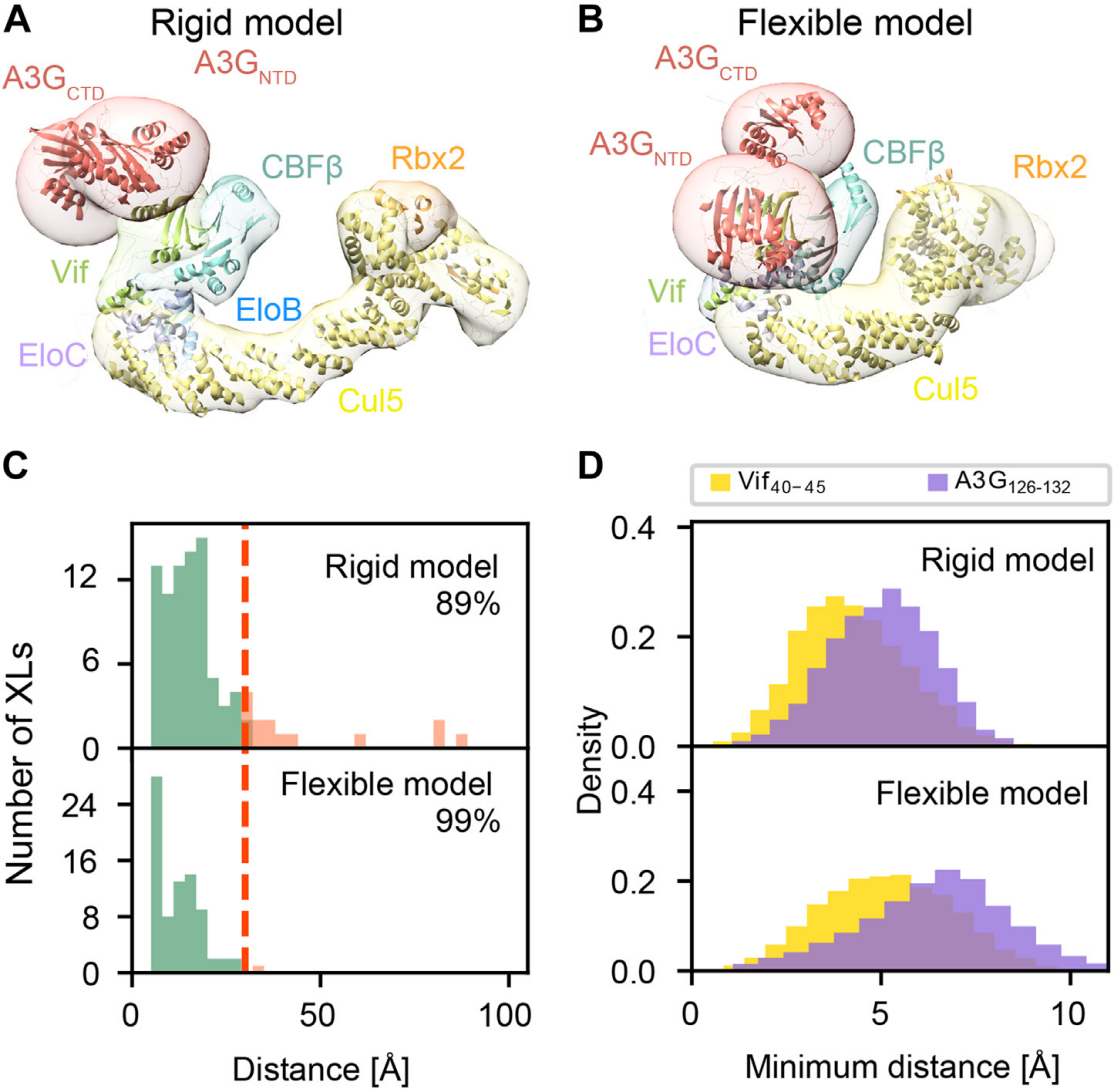
**Integrative structure of the A3G-VIf-CRL5-CBFβ complex.***A*, structure of the A3G-Vif-CRL5-CBFβ complex using a rigid representation. The localization probability density of the ensemble of structures is shown with a representative (centroid) structure from the ensemble embedded within it. A localization density map for a set of models is defined as the probability of observing a model component at any point in space. *B*, structure of the A3G-Vif-CRL5-CBFβ complex using a flexible representation. *C*, histogram showing the distribution of the cross-linked Cα–Cα distances in the A3G-Vif-CRL5-CBFβ integrative structures (*top*: rigid representation and *bottom*: flexible representation). Cross-links with Cα–Cα distances that fall within the distance threshold of 30 Å in at least one structure in the ensemble are classified as satisfied. *D*, histograms showing the minimum distances between the selected segments and the target protein surfaces for each of the protein-surface proximity restraint.

Structural models of the A3G-Vif-CLR5-CBFβ complex were obtained by exhaustive Monte Carlo sampling, starting with random initial configurations. The clustering of the ensembles identified a single cluster of models with 91% and 98% of the individual structures for the rigid and flexible models, respectively (Fig. 3, *A* and *B*). The model precision, which is defined as the average RMSD between all solutions in the ensemble, is 8.4 and 19.9 Å for the rigid (supplemental Fig. S10) and flexible model, respectively; this variability arises both from the actual structural heterogeneity and relative lack of input information for structure modeling. In the rigid model, 89% of the cross-links are satisfied by at least one model in the structural ensemble, including all of the cross-links attributed to A3G (Fig. 3*C* and supplemental Fig. S9*B*). Unsatisfied cross-links span mostly residues between the Cul5 CTD and the Vif-CBC subcomplex, indicating that the rigid representation of the system is not adequate to capture the full range of conformations in solution. In contrast, the flexible model satisfies 99% of the cross-links (Fig. 3*C* and supplemental Fig. S9*C*). Similarly, residues included in the residue–protein proximity restraint are within a threshold distance of the binding target in the rigid and flexible models (Fig. 3*D*). This uncertainty in the output model primarily reflects the structural heterogeneity of the sample, the lack of information, or a combination of both (below). These ensembles allow us to identify the A3G binding conformation and to quantify the A3G and Cul5 structural heterogeneity within the model ensembles. For most aspects the conclusions do not depend on the representation (*i.e.*, rigid or flexible) used. For example, we obtained the same A3G-Vif interface with the rigid and flexible representations (supplemental Fig. S11).

To validate the model and to determine if the data types used are consistent with each other, we computed the model using subsets of the data. For example, to determine the effect of the two residue–protein proximity restraints on both the model and its precision, we recomputed the model of the A3G-Vif-CRL5-CBFβ complex excluding these restraints using the rigid representation (supplemental Fig. S10). As expected, the resulting model is less precise (model precision of 13.7 Å), although it has a similar cross-link satisfaction (89%) than the model computed with the residue–protein proximity restraints. The model computed without the residue–protein proximity restraints has the same architecture as the original model; in particular, the A3G-Vif interface is conserved within the precision of the model (supplemental Fig. S11*B*). As another validation, we recomputed the structure including random subsets of cross-links (*i.e.*, “jackknifing”) (60) (supplemental Fig. S11). Removing a small fraction (20%) of the cross-links had no effect on the model precision, but removing larger fractions resulted in less precise models. The obtained A3G-Vif interfaces are similar to the original interface (*i.e.*, rigid model), suggesting that the cross-link data are accurate, and the models are not a result of overfitting. This result is expected when the uncertainty in the output model reflects primarily structural heterogeneity of the sample, not the lack of data. Finally, we recomputed the model without discriminating among the cross-links based on their composite confidence scores (supplemental Fig. S11). This is equivalent to just filtering the cross-links based on reproducibility and a threshold score. The resulting model has two clusters (populations of 50% and 25%), is less precise (model precisions 10.5 and 9.1 Å) than the original model, although each cluster satisfies the cross-links (89%) as well as the original model. The A3G-Vif interfaces in these clusters are similar to the original interface, indicating that cross-link composite confidence scores improves the precision of the resulting models. In conclusion, increased precision of the model resulting from using a larger number of restraints also increased our confidence in the model and the data alike (subject to sufficient sampling, SI Methods, supplemental Tables S7 and S8).

### The Structure Reveals That A3G Binds to Vif Mostly Through Its NTD

**Fig. 4:**
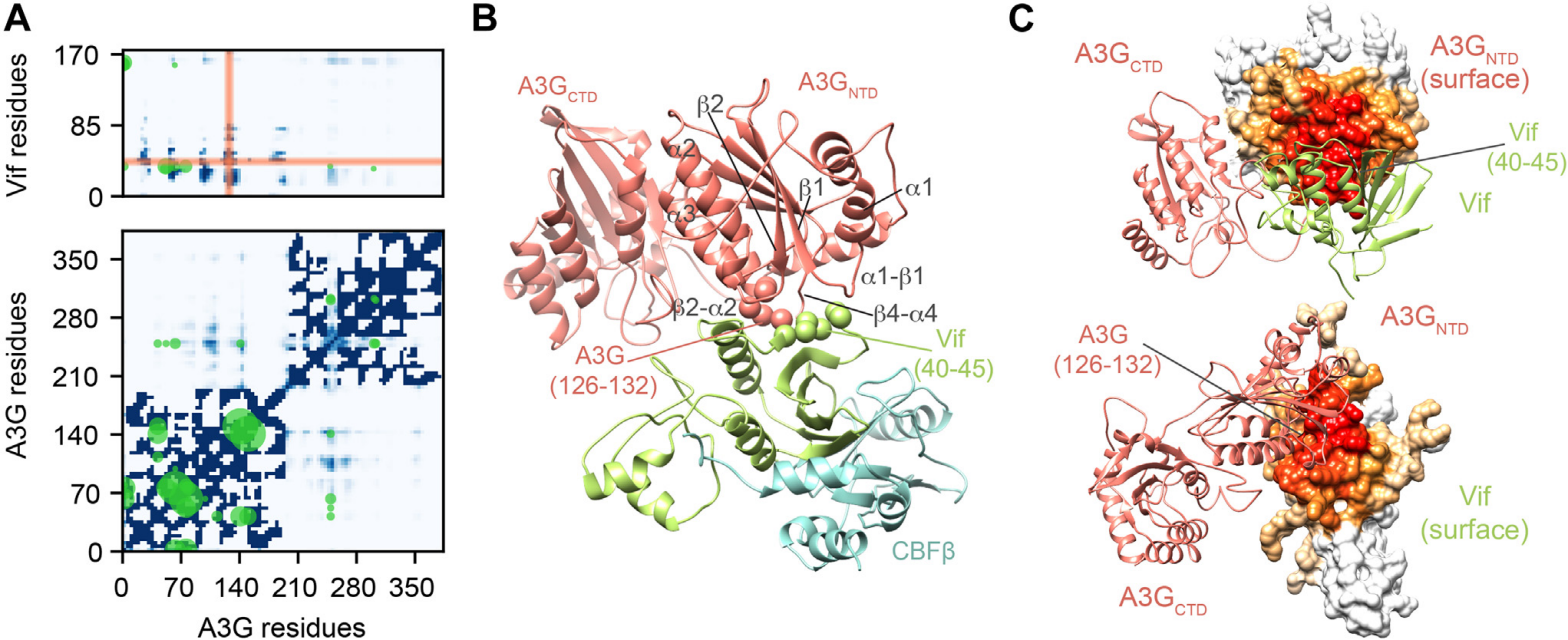
**A3G binding to the CRL5-Vif-CBFβ complex.***A*, contact maps computed for A3G and the A3G-Vif interface. The *blue bins* indicate pairs of proximal beads representing the model, with the intensity of blue proportional to the fraction of models in the cluster whose Cα–Cα distance is closer than the cutoff of 12 Å. The *green circles* correspond to the cross-links satisfied by at least one model in a cluster, with the size of the circle proportional to the spectral count. The *orange vertical* and *horizontal regions* correspond to the segments selected for the residue–protein proximity restraint. *B*, detail of the A3G -Vif binding interface. Segments A3G 126 to 132 and Vif 40 to 45 are shown as spheres. *C*, A3G-Vif binding interface. Surface representation of Vif (*top*) and A3G (*bottom*) showing the binding interface with the intensity of red proportional to the fraction of models in the cluster whose Cα–Cα distance is closer than the cutoff of 12 Å.

Normalized contact frequencies, defined by how often in the ensemble any pair of residues contact each other in the ensemble, identified a single A3G-Vif interface (Fig. 4, *A* and *C*). Mapping of the normalized contact frequencies to the protein surfaces revealed that the A3G NTD and CTD domains interact mostly through the 241 to 258 loop (Fig. 4*B* and supplemental Fig. S12*A*). The A3G-Vif interface contains the residues identified in mutagenesis-based studies, including the residues restrained by residue–protein proximity restraint (*i.e.*, A3G 126–132 and Vif 40–45) as well as other regions, such as A3G loops α1-β1, β2-α2, and β4-α4 (75) and Vif residues 22 to 26 (32) (Fig. 4*B*). In addition to these previously described A3G-Vif contacts, we also identified A3G regions 30 to 35, 55 to 66, 92 to 101, and 188 to 195 as part of the A3G-Vif interface (supplemental Table S9).

### The Integrative Structure of A3G-Vif-CRL5-CBFβ Reveals That the A3G CTD and Cul5 Subunits Are Structurally Dynamic

**Fig. 5:**
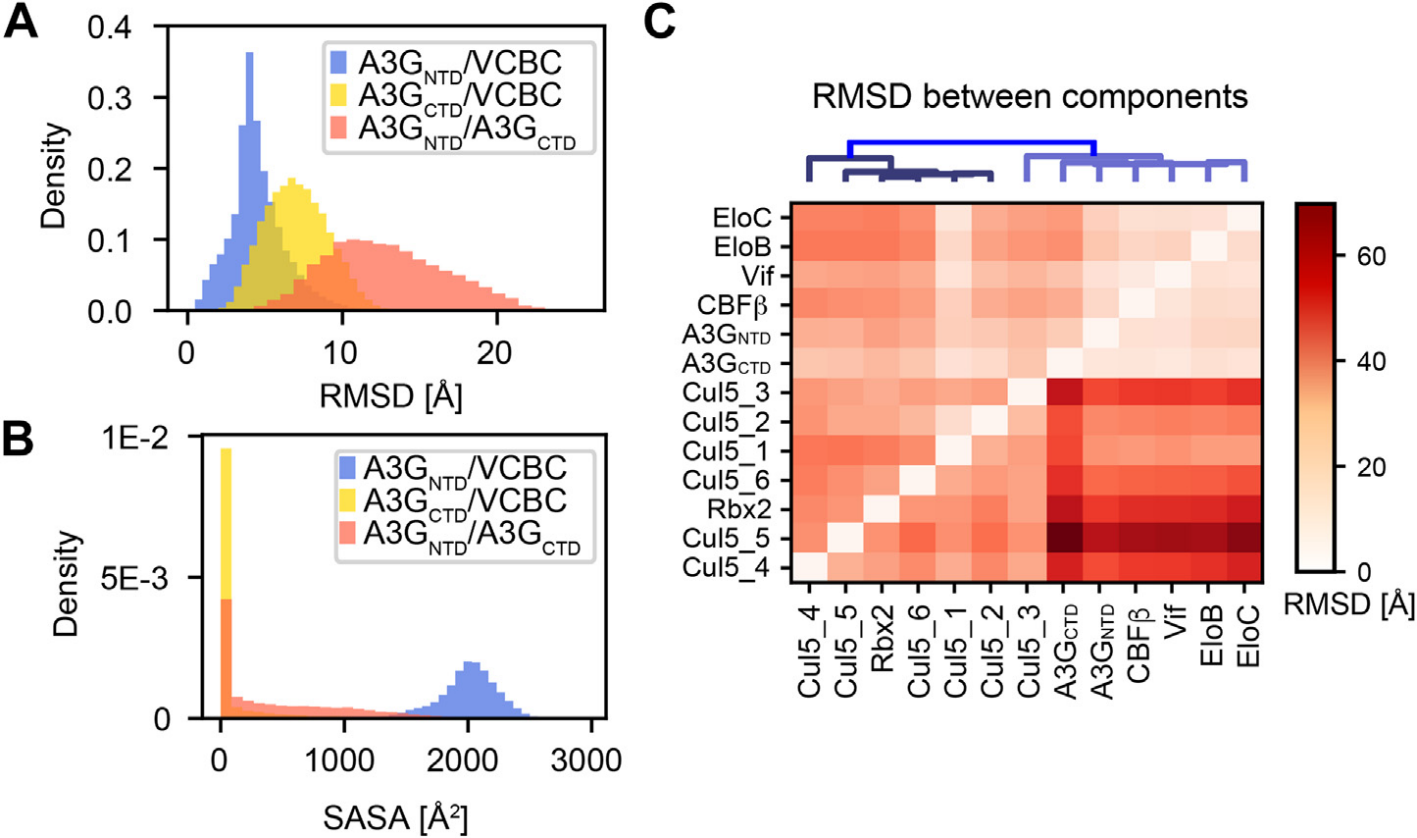
**Structural heterogeneity of the A3G-Vif-CRL5-CBFβ complex.***A*, distribution of RMSD between the A3G domains and Vif-CBC subcomplex after superimposing the Cα coordinates of Vif-CBC computed for all structures in the ensemble. *B*, distribution of SASA for the A3G NTD-Vif, A3G CTD-Vif, and A3G NTD-CTD interfaces computed for all structures in the ensemble. *C*, RMSD between rigid bodies in the model ensemble. The *vertical axis* corresponds to the rigid body used as reference for superimposition and the *horizontal axis* are the rigid bodies for which the average RMSD was computed. Cul5 segments numbered 1 to 6 correspond to the rigid bodies between the flexible linkers (supplemental Table S8).

The A3G CTD is not localized precisely in our model. We quantified the configurational and conformational heterogeneity of the A3G NTD and CTD domains using two structural metrics. First, we computed the RMSD of each domain with respect to the Vif-CBC subcomplex and with respect to each other (Fig. 5*A*). After superimposing the Cα coordinates of the Vif-CBC complex, the average RMSD of the A3G NTD and CTD domains is 4.5 ± 1.8 Å and 7.0 ± 2.0 Å, respectively. Similarly, the RMSD of the CTD domain with respect to the NTD domain is 12.8 ± 3.8 Å. Second, we computed the solvent-accessible surface area (SASA) for the A3G NTD-Vif, A3G CTD-Vif, and A3G NTD-CTD interfaces (Fig. 5*B*). Together, computed RMSDs and SASAs indicate that the A3G CTD (i) adopts a range of conformations, (ii) does not have a significant binding interface with Vif, and (iii) is only loosely associated with the A3G NTD. The A3G NTD-CTD interaction occurs mostly through the 241 to 258 loop (supplemental Fig. S12*A*). This conformation has previously been described as the dumbbell form, for free (78) and ssDNA-bound A3G (79).

In the flexible model ensemble, Cul5 adopts a closed conformation, bringing the Cul5 CTD closer to the Vif-CBC complex (Fig. 3*B*). Importantly, the proteins in the Vif-CBC-Cul5_N__TD_ subcomplex adopt configurations that are similar to the one in the X-ray structure. Configurational changes include the rotation of EloB, EloC, and Cul5 with respect to Vif (supplemental Fig. S12*B*). The average RMSD between the X-ray structure and the models of Vif-CBC-Cul5_N__TD_ in the ensemble is 12.1 Å (supplemental Fig. S12*C*).

To indicate the most flexible parts of the structure, we assessed the uncertainty of the position and orientation of each rigid body in the flexible model ensemble. To this end, all models were superimposed on each rigid body in turn, followed by computing the average RMSD for each of the other rigid bodies (Fig. 5*C*). The model ensemble indicates large variability in the positions and orientations of some of the Cul5 domains with respect to the Vif-CBC complex. In particular, the Cul5 1 to 302 region presents a lower RMSD variability with respect to the Vif-CBC complex, while a large variability is observed for the globular Cul5 CTD. This observation is consistent with the structural heterogeneity of this region indicated by the relative lack of electron density from crystallography and molecular dynamics studies that indicate the Cullins are flexible proteins with conserved hinges in the NTD (80, 81). Furthermore, it has been shown that Neddylation of Cul5 leads to a conformational change in the Cul5 CTD (82, 83). No Nedd8 was present in our sample, but we hypothesize that a similar range of conformations is available for the sample in solution.

### The A3G Structure and A3G-Vif Interface Allow Us to Rationalize Structural, Biochemical, and Functional Data

**Fig. 6:**
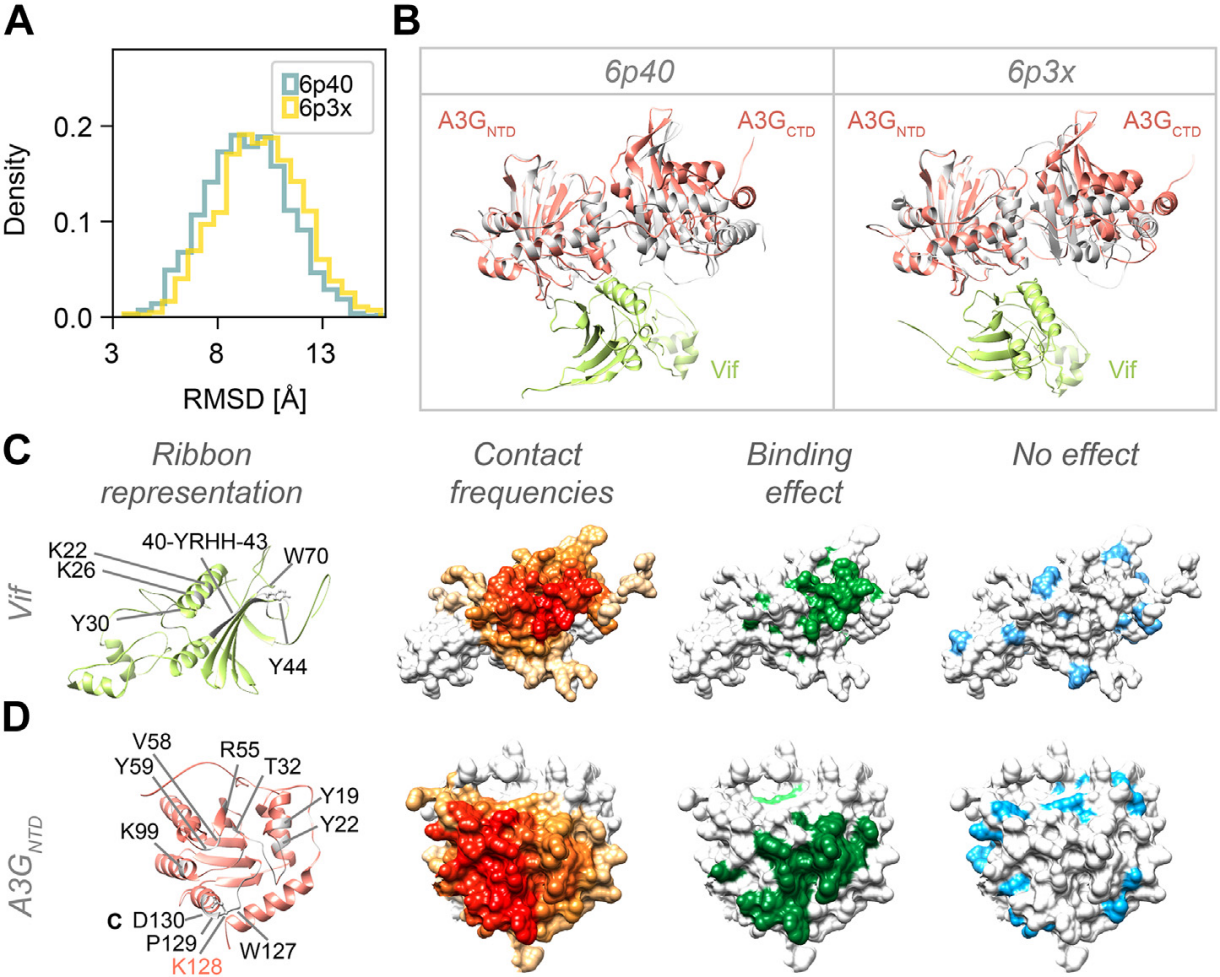
**Rationalization of the A3G structure and A3G-Vif Interface by structural, biochemical, and functional data.***A*, comparison of the A3G-bound to Vif-CRL5-CBFβ integrative ensemble to the rA3G X-ray structures (PDB codes 6p40 and 6p3x) (84). Histogram showing the Cα RMSD between A3G in all the model ensemble and the two rA3G X-ray structures. *B*, superimposition of the A3G-bound integrative (*colored*) and rA3G (*gray*) X-ray structures. The superimposition is based on the A3G NTD to show the rotation of the A3G CTD. Vif (*green*) is shown for reference. *C*, residue on Vif involved in A3G binding. From *left* to *right*: *ribbon* representation of Vif showing key residue that is known to be required for A3G-Vif binding; surface representation of Vif showing the normalized contact frequencies for the Vif-A3G NTD interface. The intensity of red is proportional to the normalized contact frequency of models in the integrative ensemble whose Cα–Cα distance is closer than the contact cutoff of 12 Å; surface representation of Vif showing in *green* the residues involved in A3G binding identified in mutagenesis studies; and surface representation of Vif showing in *cyan* the residues that had not effect on A3G binding in mutagenesis studies. *D*, residue on A3G NTD involved in Vif binding. Same as panel *C*.

We also quantified the degree to which the model satisfies relevant information not used to compute it, including data from structural, biochemical, and functional studies. First, we compared our integrative structure of A3G bound to the Vif-CRL5-CBFβ complex to the recently published X-ray structure of the full-length double-domain rhesus macaque A3G (rA3G) (84). The rA3G structure revealed that the two domains interact through a flexible linker (residue 194–198 in rhesus macaque and 195–199 in humans). However, two rA3G constructs differing in several point mutations have a different packing orientation between the two rA3G domains. To compare the X-ray structures to the integrative modeling ensemble, we computed the distribution of the Cα root-mean-square deviation (RMSD) between the X-ray structures and each of the models in the ensemble (Fig. 6*A*). The mean/minimum Cα RMSDs are 9.4/3.6 and 10.0/3.5 Å for the 6P40 and 6P3X structures, respectively (84). The Vif-CRL5-CBFβ bound A3G structure superimposes well on the rA3G structure and shares the same domain interface, mediated by the 243 to 258 flexible linker (Fig. 6*B*). The RMSD distributions show that the integrative structure ensemble includes the X-ray structures within the precision of the model and captures the overall conformational heterogeneity of the domain orientations. Thus, the integrative structure is validated by the X-ray structure, even though the X-ray structure covers only one out of seven protein subunits.

Second, we examined how the A3G and Vif regions that have been indicated as having functional effect by mutagenesis studies map to the A3G-Vif interface on the integrative model. The A3G-Vif interaction has been studied extensively, mainly through monitoring the mutation effect on A3G degradation in cells, A3G packaging into viral particles, A3G-Vif binding stability, and HIV restriction. To this end, we prepared a comprehensive overview of the A3G and Vif mutations and their functional effects (supplemental Tables S9 and S10, supplemental Fig. S13) and map these regions to the A3G-Vif interface on our integrative model (Fig. 6, *C* and *D*). Specifically, we mapped A3G and Vif mutations shown to: 1) disrupt A3G-Vif binding (21, 22, 44, 75), 2) protect A3G from degradation in cells, 3) restore packaging into the virions or virus-like particles (40, 85, 86, 87), 4) restore A3G restriction activity by hypermutation of the HIV-1 genome (40, 88). Additionally, we mapped mutations that have no demonstrated functional effect. Residues or regions where studies have seemingly contradictory data (*e.g.*, some publications assert no effect while others assert an effect) have been flagged as uncertain (supplemental Tables S9 and S10). We observe a good correlation between the A3G and Vif residues binding interface in the model and the residues known to affect binding and/or function. Similarly, we observe that “neutral” residues are scattered on the surface of the proteins and mostly do not localize to the interaction interface.

Third, we assessed whether A3G residues that have been indicated to have structural or functional effect in Vif-mediated A3G degradation localize to the A3G-Vif interface. Comparative structural analyses have determined that Vif-binding A3 domains have negatively charged regions (89) and suggest A3G negatively charged residues at α3, α4, loop 6, loop 8, β3, β4, and β5 are part of the A3G-Vif interface. Notably, negatively charged residues within this region have an average contact frequency of 68% in the integrative modeling ensemble (supplemental Table S9). Structure-guided mutagenesis studies (90) and ubiquitination analyses (91, 92) identified four A3G CTD lysines (297, 301, 303, and 334) that are implicated in Vif-dependent A3G ubiquitination and degradation. In our integrative structure, all of these lysines localize far from the A3G-Vif interface, in the flexible A3G CTD. The large dynamic range of conformations adopted by the A3G CTD may help facilitate ubiquitin transfer.

## Discussion

In this work, we present a pipeline to streamline the structure characterization of host–pathogen complexes by using integrative structure modeling based on chemical cross-link data and predicted residue–protein contacts from mutagenesis studies. This pipeline was validated by determining the structure of the A3G-Vif-CRL5-CBFβ complex. By pooling cross-linked peptides from different Vif-containing subcomplexes (Fig. 1), we identified a large number of DSSO-modified peptides, thus improving the coverage of the sequences detected by MS and the number of cross-links for each subunit. We identified 132 high-confidence cross-links, for which we assigned a composite confidence score based on their frequencies and the MS scores. This cross-link dataset, as well as atomic structures of the subunits, and highly reproducible data from mutagenesis studies, enabled us to compute the integrative structure of the entire heptameric A3G-Vif-CRL5-CBFβ complex. We show that including the cross-links composite confidence scores and the mutagenesis data improved the precision of the structural ensembles obtained using integrative structure modeling. In addition, we exemplify how integrative structures can be validated by randomly removing data and resampling to determine if the data types used are consistent with each other and to assess the sources of heterogeneity in the data. This study shows the feasibility of using DSSO-based XL-MS analysis for integrative modeling of structurally heterogeneous host–pathogen protein complexes.

The structure of the full-length A3G-Vif-CRL5-CBFβ complex indicates that Vif predominantly interacts with the A3G NTD. Although the A3G-Vif interaction interface has been characterized in multiple mutagenesis experiments (see supplemental Tables S9 and S10 for extensive review; summarized in supplemental Fig. S13), our modeling combines these biochemical and genetically identified residues with cross-link data to describe the heptameric A3G-Vif-CRL5-CBFβ. The A3G-Vif interface includes regions that have been predicted to be part of the interface by multiple mutagenesis experiments (*i.e.*, A3G residues 126–132, loops α1-β1, β2-α2, and β4-α4; and Vif 40–45 and 22–26) (21, 41, 93), as well as regions that have not previously predicted (*e.g.*, A3G: 30–35, 55–66, 92–101, and 188–195). Importantly, we obtained a model ensemble that satisfies all the input information, including 99% of the input cross-links and the distances implied by the two residue–protein proximity restraints.

Cross-linking data is derived from solution samples that may exhibit structural heterogeneity within the timescale of the experiment. The origin of heterogeneity can be dynamic or static (94, 95, 96). Whereas static heterogeneity may arise from different stable conformational states that do not interconvert between each other on the timescale of the experiment, dynamic heterogeneity corresponds to structural fluctuations of the system on the timescale of the experiment. In the case of A3G-Vif-CRL5-CBFβ, it is likely that the cross-linking data reflect the dynamic heterogeneity of the system, given the samples are in solution at 37 °C. While A3G and Cul5 are known to be flexible (78, 80) to allow for the functional ubiquitin transfer (97), our cross-link dataset and integrative model provide additional evidence to support this notion. First, violations of some cross-links by the X-ray structure of Vif-CBC-Cul5_NTD_ and comparative model of Vif-CRL5-CBFβ subcomplexes indicate that the cross-link dataset reflects either a different conformational state or a range of possible structures of the A3G-Vif-CRL5-CBFβ complex. Furthermore, integrative modeling using a rigid representation of Cul5 resulted in violations of a large number of Cul5 intra- and inter-subunit cross-links, despite thorough structural sampling. These violations imply that Cul5 adopts multiple conformations in solution. When a flexible representation of Cul5 was used, the model satisfies 99% of the cross-links. Second, our model indicates that the A3G CTD is not well localized. In integrative modeling, poor localization of a protein or domain implies a lack of information, heterogeneity in the structure, or a combination of both. Modeling using only a fraction of the cross-link dataset did not affect the predicted A3G-Vif interface or the model precision, indicating that the uncertainty in the output model reflects primarily structural heterogeneity of the sample, not the lack of data. Moreover, the A3G CTD contains seven lysines, five of which reacted with DSSO. In comparison, the A3G NTD contains 13 lysines, ten of which reacted with DSSO. Qualitatively, lysines in both A3G domains are similarly reactive to DSSO and peptides derived from these domains are similarly detected by MS. Consequently, the data indicate differences in localization of the two A3G domains, rather than a difference in their reactivity or detectability in XL-MS experiments. Lastly, we used a Bayesian scoring function (98, 99) to simultaneously model an ensemble of A3G-Vif-CRL5-CBFβ structures and infer additional parameters, such as the uncertainty in the cross-linking data. This Bayesian approach revealed that the computed data uncertainty is consistent with the independently estimated uncertainty of the experimental data (supplemental Table S10), indicating that the ensemble of models appropriately represents the system.

Distinct binding interfaces, corresponding to the different members of the A3 family (*i.e.*, A3G, A3F, or A3H), have been mapped to the N-terminal **α/β** domain of Vif. For example, A3F binds to a site involving Vif residues 11 to 17, 74 to 80, and 171 to 174 (100, 101, 102). A3H binding interface involves Vif residues 39, 48, and the 60 to 63 segment (103, 104, 105). The normalized contact frequencies indicate that Vif residues involved in binding A3F and A3G have normalized contact frequencies below 60% in the A3G-Vif-CRL5-CBFβ integrative structure. In contrast, residues involved in A3G binding have normalized contact frequencies above 90% (supplemental Table S9). These high normalized contact frequencies are observed for Vif residues used for modeling in the residue–protein binding restraint (40, 41, 42, 43, 44, 45) as well as for Vif residues whose binding information was not used for modeling (*i.e.*, Vif residues 22, 26, 30, 53, and 70). These results indicate that our integrative approach identified the binding site on Vif that binds specifically A3G, but not to other A3s.

Reactivation of A3s restriction activity is an attractive HIV-1 treatment strategy. It has been shown that disruption of the A3G-Vif-CRL5-CBFβ complexes as well as inhibition of downstream components, such as the proteasome, can restore antiviral activities and attenuate HIV infectivity (106, 107). Current therapeutic strategies targeting Vif include disrupting the Vif multimerization, A3G-Vif interface, Vif-EloC interface, Vif-CBFβ, and developing A3G up regulators (108, 109). By identifying the larger A3G-Vif interface, we might be able to design a combination strategy that prevents the virus from escaping A3G antiviral activity *via* a single point mutation.

The experimental and computational protocols described here are generally applicable to other difficult to characterize host–pathogen protein complexes. Moreover, this XL-MS and integrative modeling approach can be expanded to include other orthogonal data types, as well as data collected *in vivo*. For example, these XL-MS methods can be applied *in vivo* to infected cells for integrative structure determination of host–pathogen protein assemblies that are difficult to purify, are involved in viral-mediated signaling, localize to special cellular compartments, or are membrane associated. As the field of XL-MS moves toward intracellular applications, the potential for studying host–pathogen complexes, pathways, and networks on an unbiased level becomes more feasible. Complementary to this *in vivo* XL-MS application is the use of *in vivo* genetic interactions obtained using the point mutant epistatic miniarray profile (pE-MAP) platform to derive spatial restraints for integrative modeling (110, 111, 112). In such cases, the structure of host–pathogen complexes and how they affect infection can be studied by introducing specific mutations into the pathogenic genome and studying the phenotypic consequences using genetic interaction profiling of relevant host genes. Combining XL-MS and pE-MAP data has proven to improve the precision and accuracy of the models (111).

## Data Availability

The mass spectrometry proteomics data are available *via* ProteomeXchange with the dataset identifier PXD025391. Annotated spectra for all interlinked, loop-linked, dead-end, and single peptides can be found on the MS-Viewer application through ProteinProspector (58) (https://msviewer.ucsf.edu/prospector/cgi-bin/msform.cgi?form=msviewer) with the search key 9tjmaqhszr. All the relevant scripts, data, and results are available at GitHub, https://github.com/integrativemodeling/A3G-CRL5-Vif-CBFb. The integrative structures of A3G-Vif-CRL5-Vif-CBFβ have been deposited at PDB-Dev (https://pdb-dev.wwpdb.org/) with the accession code PDBDEV_00000090 and PDBDEV_00000091. The open-source software suite for integrative structure modeling, Integrative Modeling Platform (IMP), is available at https://integrativemodeling.org.

## Supplemental data

This article contains supplemental data (5, 7, 17, 20, 21, 22, 48, 58, 64, 71, 73, 74, 75, 76, 99, 113, 114, 115, 116, 117, 118, 119, 120, 121, 122, 123, 124, 125, 126, 127, 128, 129, 130, 131, 132, 133, 134, 135, 136, 137, 138, 139, 140, 141, 142, 143, 144, 145, 146, 147, 148, 149, 150, 151, 152, 153, 154, 155, 156, 157, 158, 159, 160, 161, 162, 163, 164, 165, 166, 167, 168, 169, 170, 171, 172, 173, 174, 175, 176, 177).

## Conflict of interest

N. J. K. has consulting agreements with the Icahn School of Medicine at Mount Sinai, New York, Maze Therapeutics, and Interline Therapeutics. He is a shareholder in Tenaya Therapeutics, Maze Therapeutics, and Interline Therapeutics.

## Supplemental Data

bt_trypsin.txt

bt_trypsin.xml

bt_chymotrypsin.txt

bt_chymotrypsin.xml

sc.txt

sc.xml

SI Methods, SI Results and Supplemental Figures S1–S13

Supplemental Table S10

PA.SwissProt.PN.RMK.A3GVifCRL5.fasta.random.concat

PN.RMK_LAIVif_HisCBFB.fasta

SwissProt.Human.2016.01.11.fasta.random.concat
