# Supplementary material for: Characterization of an A3G-VifHIV-1-CRL5-CBFβ Structure Using a Cross-linking Mass Spectrometry Pipeline for Integrative Modeling of Host–Pathogen Complexes
Source: Mol Cell Proteomics. 2021 Aug 11;20:100132. doi: 10.1016/j.mcpro.2021.100132 (PMC8459920; doi:10.1016/j.mcpro.2021.100132)
Supplement: SI Methods, SI Results and Supplemental Figures S1–S13 [file mmc7.pdf]

**Supporting Information: Characterization of a A3G-Vif$_{HIV-1}$-CRL5-CBFβ structure using a cross-linking mass spectrometry pipeline for integrative modeling of host-pathogen complexes**

**Authors:** Robyn M. Kaake[1,2,4,#], Ignacia Echeverria[1,3,#], Seung Joong Kim[3,9], John Von Dollen[1,2], Nicholas M. Chesarino[5], Yuqing Feng[6], Clinton Yu[7], Hai Ta[8], Linda Chelico[6], Lan Huang[8], John Gross[2,8], Andrej Sali[2,4,8,*], Nevan J. Krogan[1,2,4,*]

[1] Department of Cellular and Molecular Pharmacology, California Institute for Quantitative Biosciences, University of California, San Francisco, San Francisco, CA 94158, USA.
[2] Quantitative Biosciences Institute, University of California, San Francisco, San Francisco, CA 94158, USA.
[3] Gladstone Institute of Data Science and Biotechnology, J. David Gladstone Institutes, San Francisco, CA 94158, USA.
[4] Department of Bioengineering and Therapeutic Sciences, University of California, San Francisco, San Francisco, CA 94158, USA.
[5] Divisions of Human Biology and Basic Sciences, Fred Hutchinson Cancer Research Center, 1100 Fairview Ave N, Mailstop C2-023, Seattle, WA, 98109, USA.
[6] Department of Biochemistry, Microbiology, Immunology, University of Saskatchewan, Saskatoon, Saskatchewan, S7N 5E5, Canada.
[7] Department of Physiology & Biophysics, University of California, Irvine, CA 92697, USA
[8] Department of Pharmaceutical Chemistry, University of California, San Francisco, San Francisco, California, USA.

[#] Contributed equally
[*] **Corresponding authors:**
Nevan J. Krogan
Quantitative Biosciences Institute, University of California, San Francisco, San Francisco, CA 94158, USA
Email: nevan.krogan@ucsf.edu

Andrej Sali
Department of Bioengineering and Therapeutic Sciences, Department of Pharmaceutical Chemistry, California Institute for Quantitative Biosciences, University of California, San Francisco, San Francisco, CA 94158, USA
Email: sali@salilab.org

This supplemental file includes:
Supplemental methods
Supplemental results
Supplemental Figures S1 to S12
Supplemental Tables legends S1 to S10

## SUPPLEMENTAL METHODS

*Reagents.*

Disuccinimidyl sulfoxide (DSSO) was synthesized as reported(17), and purchased from Thermo Fisher Scientific (Rockford, IL). Criterion TGX 4-20% SDS-PAGE gels were purchased from BioRad (Hercules, California). AcquaStain Protein Gel Stain was purchased from Bulldog Bio (Portsmouth, NH). Sequencing grade Trypsin and Chymotrypsin were purchased from Promega Corp (Madison, WI). OMIX C18 desalting tips were purchased from Agilent Technologies (Santa Clara, CA). Protein LoBind and Maxymum Recovery Snaplock Microcentrifuge tubes were purchased from Eppendorf (Hauppauge, NY) and Axygen (Gaithersburg, MD), respectively. TransIT-293 was purchased from Mirus (Madison, WI). Antibodies were purchased from Sigma: αFLAG, αp24, αGAPDH, or the AIDS resource αVif (809). All other general chemicals for buffers were purchased from Fisher Scientific or Sigma Aldrich (St. Louis, MO).

*Analysis of cross-linked peptides by LC-$MS^3$.*

*Identification of Cross-linked Peptides.* Our cross-link peptide identification framework is a stepwise process, as described (**Fig. S3**):

Step 1 - Convert and Search Raw Data: Identify XL-Remnant Modified Peptides. Each technical replicate LC-$MS^3$ run results in a single raw file (e.g., filename.raw), which is converted to mgf format (e.g., filename.mgf) using free open software MSconvert (http://proteowizard.sourceforge.net/tools.shtml) (6), both at the $MS^2$ and $MS^3$ levels individually (e.g., filename_MS2.mgf and filename_MS3.mgf). The $MS^3$ data is searched by the Batch-Tag software of a locally installed version of Protein Prospector (v. 5.19.1, University of California San Francisco) and peptide reports generated using Protein Prospector's Search-Compare feature (http://prospector.ucsf.edu). This is done in a semi-automated fashion where *Batch-tag* and *Search-Compare* input files are automatically created from a template. Supplementary files bt_trypsin.txt, bt_chymotrypsin.txt, bt_trypsin.xml, and bt_chymotrypsin.xml include description and search parameters for including missed cleavages, precursor and fragment mass tolerance, constant and variable modifications. Supplementary files sc.txt and sc.xml include the description and filtering criteria for reporting peptides including peptide and protein score and expectation value thresholds. All DSSO-remnant modifications were included and are available in a supplemental file (usermod_xlink.txt). Data files were searched against two databases: the fully annotated human proteome downloaded from Uniprot concatenated with an equal length randomized polypeptide sequence database (see provided text file with fasta formatted entries: SwissProt.Human.2016.01.11.fasta.random.concat), and a database with the affinity tagged full-length and truncated versions the A3G-Vif-CRL5-CBFβ or A3G-$V_{LAI}$CBC subunits (see provided files with protein entries in each respective database: PA.SwissProt.PN.RMK.A3GVifCRL5.fasta.random.concat and PN.RMK_LAIVif_HisCBFB.fasta). Randomized entries are used for peptide and cross-link FDR estimation. To view the annotated $MS^3$ spectra for all single, dead-end, loop-linked and interlinked peptides (α and β separated), use the MSViewer application through ProteinProspector (58) (https://msviewer.ucsf.edu/prospector/cgi-bin/msform.cgi?form=msviewer) with the search key 9tjmaqhszr.

Step 2 - Calculate Masses, Determine and Classify Cross-linked Peptides. The $MS^3$ results (e.g., filename_MS3-results.txt) files are processed in batch to uniformly format files to be readable by the in-house software XLTools, a revised version of the previously developed XL-Discoverer. XLTools was then used to batch process the cleaned files (e.g., filename_MS3-results-cleaned.txt) and validate and summarize cross-linked peptides based on $MS^n$ data and database searching (**Tables S3-S5**). XLTools reports all identified cross-linked peptides (i.e., dead-end, loop-linked, inter-linked, and singles) with their Protein Prospector scores and peptide identifying information (**Tables S3-S5**).

Step 3 - Identify Unique Inter- and Intra-Subunit Linkages, Quantify Spectral Counts, and Assign Confidence Scores for Integrative Modeling. Integrative modeling utilizes inter-linked and loop-linked peptide data, with dead-ends not contributing to the model. Therefore, once cross-linked peptides are identified, the inter-linked and loop-linked datasets (**Tables S3-S4**) are summarized and processed for integrative modeling. This entails collapsing peptides that have the same site-specific cross-links into a single entry and distributing ambiguous cross-link site assignment. First, we identify all the unique K-K and N-terminal (Nterm)-K pairs provided in the data and provide each linkage a unique identifier. A fraction of the unique K-K and Nterm-K pairs (i.e., unique linkages) identified in the data are assigned to exactly one set of unique cross-linked peptide sequence pairs (i.e., unique cross-linked peptide). The remaining K-K and Nterm-K pairs can be assigned to more than one unique cross-linked peptide sequence pair; these are the result of missed cleavages or additional post-translational modifications.

For each cross-linked peptide all identification events are summed to obtain quantified the peptide redundant count (Npep). For linkages where linked lysines are unambiguously assigned the unambiguous redundant count (i.e., Nlink) is the sum of all Npep of any contributing non-ambiguous unique cross-linked peptide (**eq. 1**).

**Eq. 1 Nlink = Σ(Npep1-n)**

For unique linkages that are the result of one or more ambiguously assigned unique cross-linked peptide (a peptide in which the modified lysine(s) could not be definitively assigned) the counts were uniquely distributed based on the XL-remnant assignments, and proportionally added to the unique residue-residue linkage events count. The distributed redundant count (dNlink) is computed as follows:

**Eq. 2 dNlink1 = Σ(Npep1-n) + Σ(Npep-ambg(Nlink1)/(Nlink1 + … Nlinkn))1-n**

Unique linkages with dNlink < 2 were filtered out and not used for integrative modeling.

Finally, each inter-linked peptide has two scores assigned to it, one for each peptide identified (provided by ProteinProspector; higher numbers indicate higher confidence in identification). For each identified peptide, the average and minimum scores were determined. For each unique linkage, the best average (Best Ave) and best minimum (Best Min) of all contributing peptide pairs is used to assign a confidence score (**Table S2**). The composite confidence score is set in three tiers: (1) High Confidence (0.01), Best Ave ≥ 25.0, or Best Ave ≥ 15 and dNlink ≥ 15; (2) Medium Confidence (0.1), 15 ≥ Best Ave < 25 and dNlink < 15 or Best Ave ≥ 15 and Number dNlink ≥ 15; and (3) Low Confidence (0.5), Best Ave < 15 and dNlink<15 (**Table S2**). These composite scores were used for integrative modeling.

In addition to the scored and filtered cross-linked dataset, the pipeline outputs visualizations of cross-linked peptide coverage for all proteins, cross-link positions, and redundant counts (**Fig. 2**), and input files for integrative modeling. All input and result files used for the analysis can be found at proteomeXchange (dataset identifier PXD025391). Scripts to generate the output files can be found at https://github.com/integrativemodeling/A3G-CRL5-Vif-CBFb.

*Integrative structure determination of the A3G-Vif-CRL5-CBFβ complex.* Integrative structure determination proceeded through the standard four stages (7, 74, 113–116) (**Fig. S8, Tables S7-S8**): (1) gathering data, (2) representing subunits and translating data into spatial restraints, (3) configurational sampling to produce an ensemble of structures that satisfies the restraints, and (4) analyzing and validating the ensemble structures and data. The integrative structure modeling protocol (i.e., stages 2, 3, and 4) was scripted using the *Python Modeling Interface* (PMI) package, a library for modeling macromolecular complexes based on our open-source *Integrative Modeling Platform* (IMP) package (115), version 2.8 (https://integrativemodeling.org). Files containing the input data, scripts, and output results are available at https://github.com/salilab/A3G-CRL5-Vif-CBFb and the nascent integrative modeling section of the worldwide Protein Data Bank (wwPDB) PDB-Dev repository for integrative structures and corresponding data (pdb-dev.wwpdb.org)(117).

Stage 1 - Gathering data. A comparative model of the human A3G NTD was computed based on the atomic X-ray structure of the primate A3G (70% sequence identity; PDB access code 5K81) (118), using MODELLER v9.20 (73, 119). A comparative model of the human A3G CTD was computed based on the atomic X-ray structure of the human A3G (97% sequence identity; PDB access codes 3V4K) (120). The atomic structure of the Vif-CBC-Cul5$_{NTD}$ pentameric complex was extracted from PDB 4N9F (48). This structure only contains the Cul5 NTD (residues 12-320). A comparative model of the Cul5 CTD in complex with Rbx2 was computed based on the atomic X-ray structure of Cul1-Rbx1 complex (PDB access code 1LDJ(121)). We used the 132 unique DSSO cross-links of the A3G-Vif-CRL5- CBFβ complex.

Stage 2 - Representing subunits and translating data into spatial restraints. To maximize computational efficiency while avoiding using too coarse a representation, we represented the A3G-Vif-CRL5-CBFβ complex in a multi-scale fashion. In particular, the domains of the A3G-Vif-CRL5-CBFβ complex subunits were coarse-grained using beads of varying sizes (i.e., 1 residue per bead and 10 residues per bead) representing either a rigid body or a flexible string, based on the available crystallographic structures and comparative models, as follows:

*(i) Rigid body definitions.* We used different rigid body definitions to address different aspects of the structure and/or configurational heterogeneity of the Vif-CRL5-CBFβ complex (**Tables S7-S8**). We defined a *'rigid representation'* to determine the structure of A3G bound to the Vif-CRL5-CBFβ complex. In this representation the atomic structure of the Vif-CBC-Cul5$_{NTD}$ pentameric complex, including the comparative model of the Cul5 CTD and Rbx2, was represented as a single rigid body. A3G was represented as two rigid bodies corresponding to the NTD and CTD regions. The linker between the A3G domains (residues 195-200) and regions missing in the X-ray structure were represented by flexible strings of beads at a one residue per bead resolution. We defined a '*flexible representation*' in which the configurations of all proteins and the conformations of A3G and Cul5 were flexible. Proteins EloB, EloC, Vif, CBFβ, Rbx2 were represented as a single rigid body each. Cul5 was represented as six rigid bodies connected by flexible linkers of 4 to 23 residues each. Hinge regions included: the loop between the second and third repeat of the NTD, the loop between the elongated NTD and globular CTD, and loop regions between the 4HB, α/β, WH-A and WH-B domains on the Cul5 CTD (**Fig. S9A, Table S8**)(76). A3G was represented as two rigid bodies as previously described.

*(ii) Scoring function.* With this representation in hand, we next encoded the spatial restraints into a Bayesian scoring function (99) based on the information gathered in Stage 1, as follows:

(1) *Cross-link restraints:* We used the 132 DSSO cross-links to construct a Bayesian scoring function that restrains the distances spanned by the cross-linked residues (5). The cross-link restraint was applied to the fine scale representation for the atomic structures and comparative models as well as to flexible beads.

(2) *Excluded volume restraints:* We applied the excluded volume restraint to each 10-residue bead, using the statistical relationship between the volume and the number of residues that it covered (122, 123).

(3) *Sequence connectivity restraints:* We applied the sequence connectivity restraint, using a harmonic upper bound on the distance between consecutive beads in a subunit, with a threshold distance equal to four times the sum of the radii of the two connected beads. The bead radius was calculated from the excluded volume of the corresponding bead, assuming standard protein density.

(4) Residue-protein proximity restraints: We used a residue-protein proximity restraint to encode information from mutagenesis studies that indicate interface residues. These studies usually detect mutations in a prey protein that disrupt the interaction with a bait protein. Mutagenesis data was converted into an upper bound on the distance between the residues identified to be required for binding and the closest residues in the predicted bound protein. This restraint was applied between A3G residues 126-132 and Vif, and Vif residues 40-45 and A3G. These restraints were derived from already published data (21, 22).

(5) Structural equivalence restraints: To encode template structure information from the Vif-CRL5-CBFβ atomic structure, we imposed distance restraints between residue pairs in distinct rigid bodies with a distance lower than 8.0 Å. The scoring function restraints the distance spanned by interface residues using a Gaussian likelihood function. The distance corresponding to the center of the Gaussian distribution was derived from the initial X-ray structure or comparative model. This restraint was applied to all proteins except A3G.

Stage 3 - Configurational sampling to produce an ensemble of structures that satisfy the restraints. The initial positions and orientations of rigid bodies and flexible beads were randomized. The generation of structural models was performed using Replica Exchange Gibbs sampling, based on the Metropolis Monte Carlo (MC) algorithm (5, 124). Each MC step consisted of a series of random transformations (i.e., rotation and translation) of the positions of the flexible beads and rigid bodies. Details about the Monte Carlo runs for each system are in **Tables S7** and **S8**.

Stage 4 - Analyzing and validating the ensemble structures and data. Model validation follows five major steps (20, 64): (i) selection of the models for validation; (ii) estimation of sampling precision; (iii) estimation of model precision, (iv) quantification of the degree to which a model satisfies the information used to compute it, and (v) satisfaction of data and considerations that were not used to compute the structures. These validations are based on the nascent Worldwide PDB (wwPDB; the global organization responsible for maintaining the PDB archive; http://www.wwpdb.org/) effort on archival, validation, and dissemination of integrative structures (117, 125). We now discuss each one of these validations in turn.

(i) *Selection of models for validation:* The first step is to objectively define the ensemble of models that will be further analyzed. For each trajectory, we automatically determined the

MC step at which all data likelihoods and priors have equilibrated (run equilibration step); and all prior frames are discarded (126). Discarding the initial, non-equilibrated steps of each run is helpful because non-typical early configurations (*eg,* a random configuration of beads, an extended configuration of beads, and beads far apart from each other) are removed from the statistical sample used for posterior model estimates. With this ensemble of sampled structures and their corresponding scores in hand, we analyze the data likelihoods and priors. We used HDBSCAN clustering, a hierarchical density-based clustering algorithm, to identify all high-density regions in the likelihoods and priors (127). If a single cluster was identified, we consider all the models after discarding the initial steps; otherwise, we consider all models in the clusters that satisfy the input information, within the uncertainty of the data, for further analysis (below).

*(ii) Estimation of sampling precision:* Next, we estimate the precision at which sampling sampled the selected structures (sampling precision) (64). As a proxy for testing the thoroughness of sampling, we performed four sampling convergence tests: 1) verify that the scores of refined structures do not continue to improve as more structures are computed, 2) confirm that the selected structures in independent sets of sampling runs (Sample A and Sample B) satisfy the data equally well, 3) cluster the structural models and determine the sampling precision at which the structural features can be interpreted (**Fig. S10**), and 4) compare the localization probability density maps for each protein obtained from independent sets of runs. Details about all the tests are described in ref. (64). For each modeling instance (i.e., rigid and flexible model), the results from the convergence tests are summarized in **Tables S7-8** and **Fig. S10**.

*(iii) Estimation of model precision*: In the third step, model uncertainty (precision) is estimated. The most explicit description of model uncertainty is provided by the set of all models that are sufficiently consistent with the input information (i.e.*,* the ensemble). Model precision can be quantified by the variability among the models in the ensemble; in the end, the ensemble can be described by one or more representative models and their uncertainties. For example, if the structures of the ensemble are clustered into a single cluster, the model precision is defined as the RMSD between models in the cluster. Importantly, the uncertainty may not be distributed evenly across the ensemble, such that some regions are determined at a higher precision than others.

*(iv) Quantification of the degree to which a model satisfies the data used to compute it:* An accurate structure needs to satisfy the input information used to compute it; all structures at computed precision that are consistent with the data are provided in the ensemble. A DSSO cross-link restraint is satisfied by a cluster of structures if the corresponding Cα–Cα distance in any of the structures in the cluster is less than 30 Å (71). The remainder of the restraints are harmonic, with a specified standard deviation. Therefore, a restraint is satisfied by a cluster of structures if the restrained distance in any structure in the cluster is violated by less than 3 standard deviations, specified for the restraint. **Tables S7** and **S8** show that all models satisfy the input information within its uncertainty.

*(v) Satisfaction of data and considerations that were not used to compute the structures.* The most direct test of a modelled structure is by comparing it to the data that were not used to compute it (a generalization of cross-validation). Our current A3G-Vif-CRL5-CBFβ structure is consistent with previously published data. A comprehensive overview of the A3G and Vif mutations and their functional effects used to validate the A3G-Vif interface on our integrative model is in (**Tables S9-S10, Fig. S11**)

## SUPPLEMENTAL RESULTS

A high proportion (47%) of DSSO modified peptides involve Cul5 residues, the largest component of the complex. Cul5 acts as a scaffold protein, binding at its NTD to the adaptor proteins (i.e., the EloB/C heterodimer), and at its CTD to the RING component of the complex (i.e., Rbx2) (**Fig. S1**) (128, 129). As such, it is not surprising that the majority of the Cul5 inter-subunit linkages were identified between the Cul5 NTD and EloC and the Cul5 CTD and Rbx2 (**Fig. 2C** and **Fig. S5**). As expected, based on the crystal structure (PDB: 4N9F), Vif cross-links to A3G, Cul5, and CBFβ (**Fig. 2C, Fig. S5**). The cross-links between Vif and Cul5 are mostly attributed to cross-linked peptides between Vif residue K122 and Cul5 residue K56. This observation is expected based on the known structure, with Vif residue K122 centered at the 100-142 region upstream of the BC-box and known to selectively bind Cul5 near the HCCH domain (130) (**Fig. S6A**). For CBFβ, we identify a total of 1179 cross-linked peptides with the vast majority spanning a residue from Cul5 and a residue from CBFβ or EloC/EloB (**Fig. 2C** and **Fig. S5**). Somewhat unexpectedly, 50% (529) of CBFβ inter-subunit linkages span residues between CBFβ (K11) and Cul5 (K126 or K127). While CBFβ has previously been documented to directly contact Cul5, the Cul5 K126 and K127 residues are missing from the X-ray crystal structure density (PDB: 4N9F), suggesting a structurally heterogeneous or dynamic region (**Fig. S6B**). 1752 DSSO cross-linked peptides involve A3G peptides, with the majority being dead-end (56%) and intra-subunit linkages (38%). Notably the only identified inter-subunit linkages are between A3G and Vif (**Fig 2C, Fig. S5**). In total, 6 residues from the A3G NTD (M1, K2, K52, K63, K76, K99) and 2 residues of the CTD (K249, K301) form cross-links to Vif, with 94% of the A3G-Vif cross-links mapping to the A3G NTD. A higher proportion of the modified lysines in the NTD form intra-subunit inter-links while most CTD lysines are modified by dead-ends. This observation is in agreement with several mutagenesis-based studies that indicate the A3G NTD is primarily responsible for Vif binding, while the A3G CTD remains flexible in solution.

**Supplemental figures and captions**

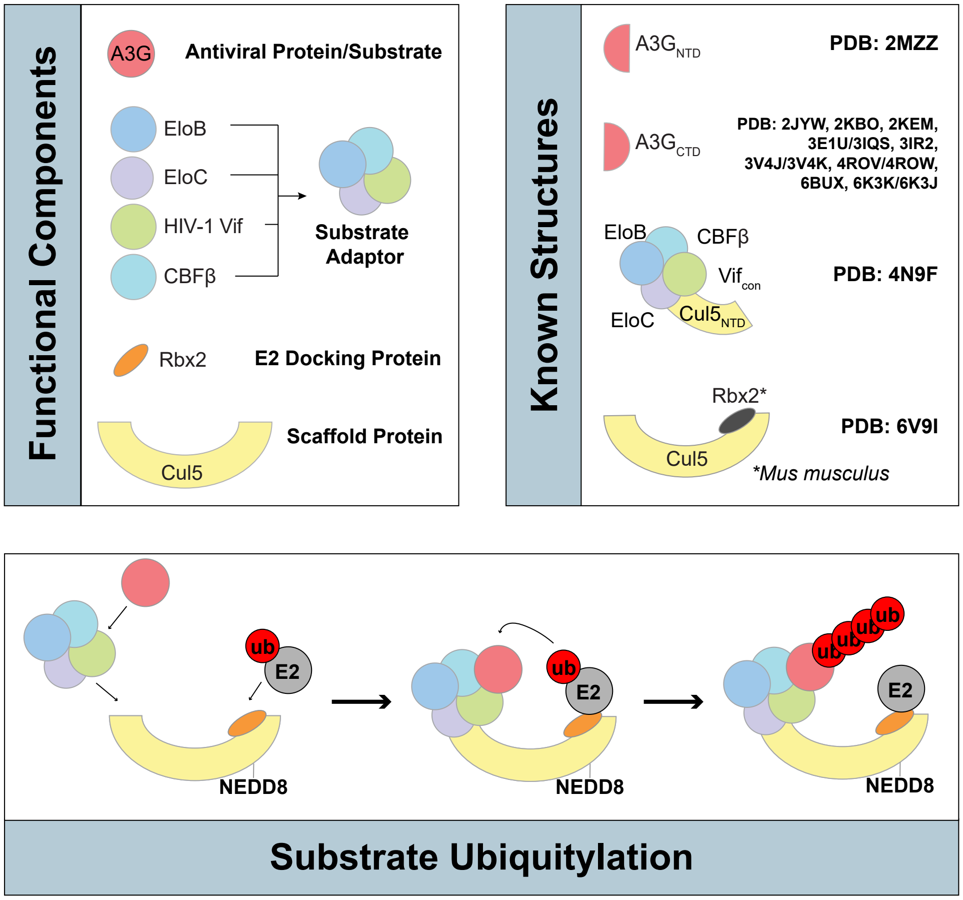


**Figure S1. Cullin 5 (Cul5)-RING E3 Ubiquitin Ligase (CRL5).** Cartoon representation of the main components of the functional CRL5 complex which include the Cul5 scaffold protein and the Rbx2 E2 docking protein, as well as the EloB/EloC substrate adaptor subcomplex that is hijacked by HIV-1 Vif. Host transcription factor CBFβ binds to HIV-1 Vif and stabilizes the substrate adaptor complex. The antiviral APOBEC3G (A3G) host protein is the target substrate that binds HIV-1 Vif and is poly-ubiquitylated in an iterative cycle. Shown on the upper left panel are the cartoon representations of known structures of the functional A3G-Vif-CRL5-CBFβ complex including the A3G NTD (75), A3G CTD (120, 128–135), Vif-CBC-Cul5$_{NTD}$ (48), and human Cul5 bound to mouse Rbx2 (136). In the bottom panel is a cartoon diagram depicting the active NEDD8 conjugated Cul5 bound to Rbx2 and an E2 protein. The Vif-CBFβ heterodimer binds to A3G and the EloB-EloC heterodimer. The active complex carries out subsequent rounds of ATP-dependent ubiquitylation of A3G, and the resulting polyubiquitylated A3G is released and degraded by the 26S proteasome.

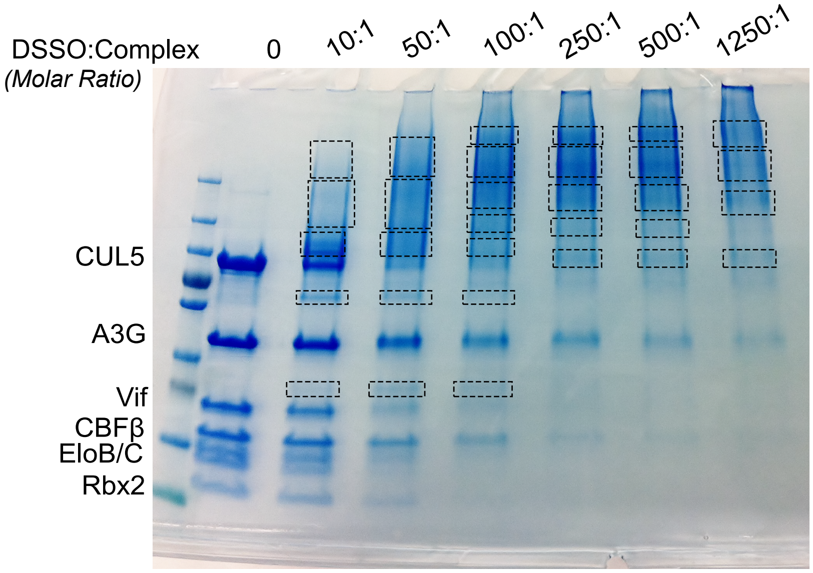


**Figure S2. Representative blue stained SDS-PAGE separation of DSSO cross-linked A3G-Vif-CRL5-CBFβ complex.** Each of the A3G-Vif-CRL5-CBFβ, Vif-CRL5-CBFβ, Vif-CBC-Cul5$_{NTD}$, and A3G-V$_{LAI}$-CBC subcomplexes were cross-linked by DSSO. Representative image of A3G-Vif-CRL5-CBFβ cross-linked with increasing molar ratios of DSSO, separated on 4-20% TGX SDS-PAGE gel, and stained with MS safe blue stain. Cross-linked products were excised from the gel (black dashed boxes) and digested for XL-MS$^3$ analysis.

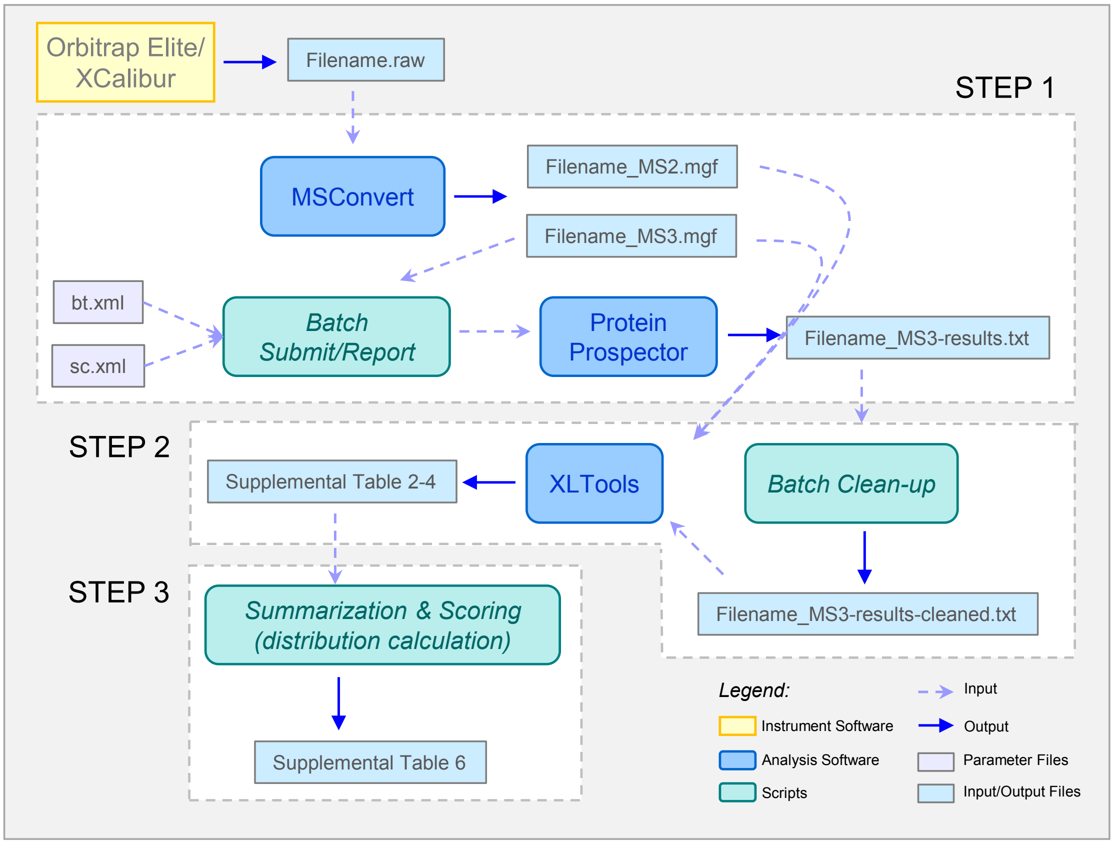


**Figure S3. Cross-link peptide identification, counts distribution, quantification and scoring pipeline.** Peptide samples are separated and analyzed by LC-$MS^3$ on a Thermo Fisher Scientific Orbitrap Elite and *.raw files produced by Xcalibur software. $MS^2$ and $MS^3$ data are extracted and converted to separate .mgf files by MSConvert (40). Scripts for automated batch submission and report generation are used to create the parameter files for BatchTag and SearchCompare features of ProteinProspector (41) from a bt.xml and sc.xml template. MS3.mgf files are searched by a locally installed version of Protein Prospector (v. 5.19.1, University of California San Francisco) and the *–results.txt files reformatted. MS2.mgf, MS3.mgf, and cleaned MS3-results.txt files are input into XLTools for cross-linked peptide identification (including the identification of dead-end, loop-linked, inter-linked, and single peptides (**Tables S2-S4**). Inter-linked and loop-linked data is quantified using the distributed spectral count wherein each unique linkage is counted across all runs and represented by a best Average Score and best Minimum Score. Data is filtered to produce **Table S6**. In the figure, Filename is used to represent the dataset which is summarized in the metadata file provided as **Table S1**.

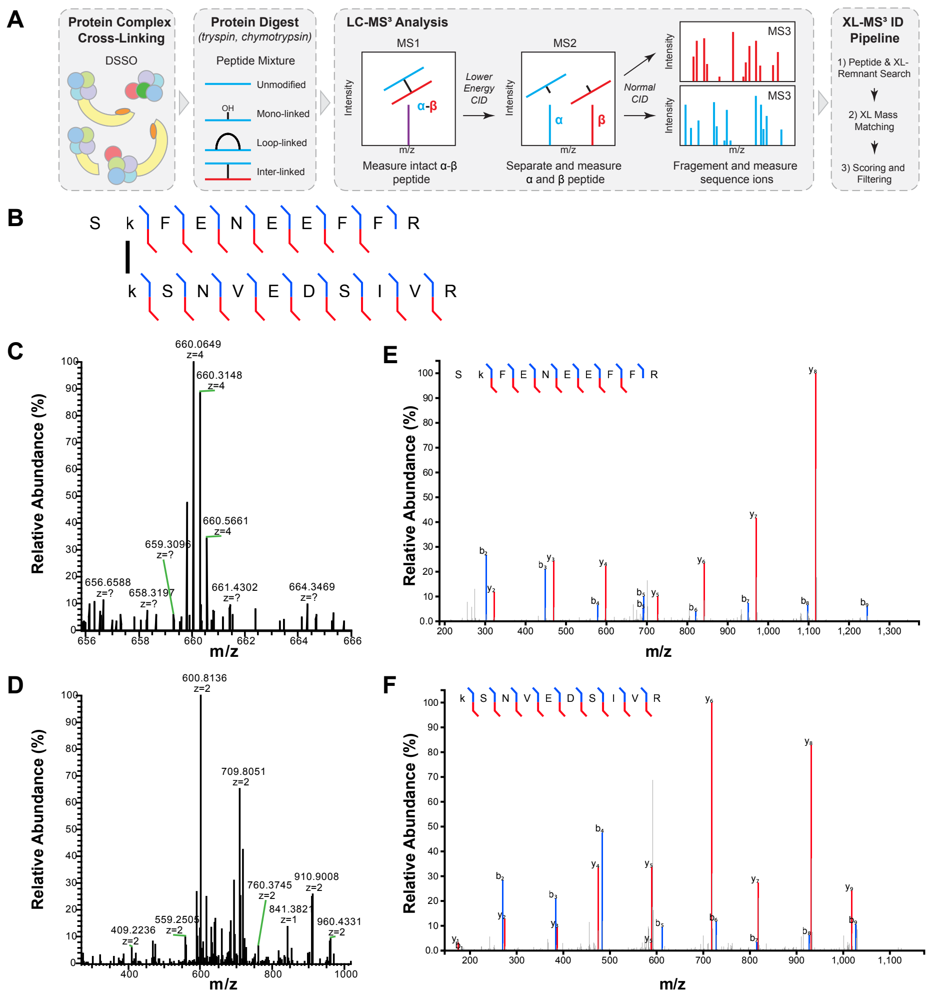


**Figure S4. DSSO-based XL-MS$^3$ strategy and representative MS$^{1-3}$ Spectra. A.** Overview of the DSSO XL-MS³ analysis method for representative Cul5 peptide KSNVEDSIVR cross-linked at K127 to CBFβ peptide SKFENEEFFR cross-linked at K11. **B.** Cartoon representation of cross-linked peptide b/y ion coverage. **C.** MS¹ spectra of intact cross-linked peptide (m/z=659.8148;

charge=4+). **D.** $MS^2$ spectra indicating α-alkene peptide species ($K_{Alkene}$SNVEDSIVR, m/z=600.84146; charge=2+) and β-thiol peptide species (S$K_{Thiol}$FENEEFFR; m/z=709.8059; charge=2+). E. $MS^3$ spectra for $K_{Alkene}$SNVEDSIVR peptide. F. $MS^3$ spectra for S$K_{Thiol}$FENEEFFR peptide. The alkene modification (alkene exact mass= 54.01056 Da) results from one half of the DSSO cross-linker cleavage in the collision cell. The thiol (unsaturated thiol exact mass= 85.98264) modification is produced by a reaction in the instrument after cleavage in the collision cell of the DSSO linker to a sulfenic acid.

**A**

Inter-Linked Peptides

| | A3G | Vif | CBFB | EloB | EloC | Cul5 | Rbx2 |
|---|---|---|---|---|---|---|---|
| A3G | 327 | 96 | 0 | 0 | 0 | 0 | 0 |
| Vif | 96 | 591 | 12 | 0 | 0 | 93 | 1 |
| BFB | 0 | 12 | 9 | 166 | 312 | 558 | 0 |
| EloB | 0 | 0 | 166 | 97 | 428 | 0 | 0 |
| EloC | 0 | 0 | 312 | 428 | 27 | 883 | 0 |
| Cul5 | 0 | 93 | 558 | 0 | 883 | 2299 | 189 |
| Rbx2 | 0 | 1 | 0 | 0 | 0 | 189 | 11 |

0 Max

**B**

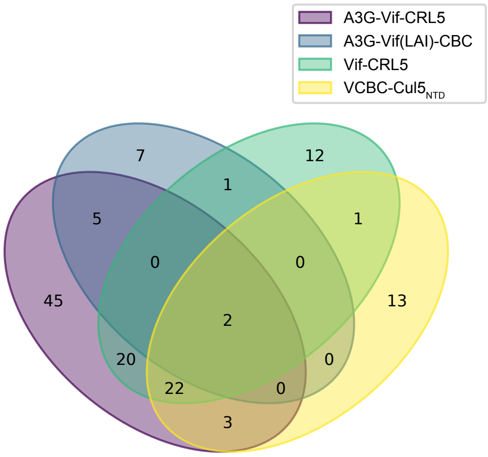


**Figure S5. Summarization of cross-linked peptides by subunit and number of unique K-K linkages by Vif-containing subcomplex. A.** Inter-linked peptides are summarized in the table by their redundant count. **B.** Venn-diagram showing the number of unique K-K linkages identified for each Vif-containing sub-complex.

**A**

**B**

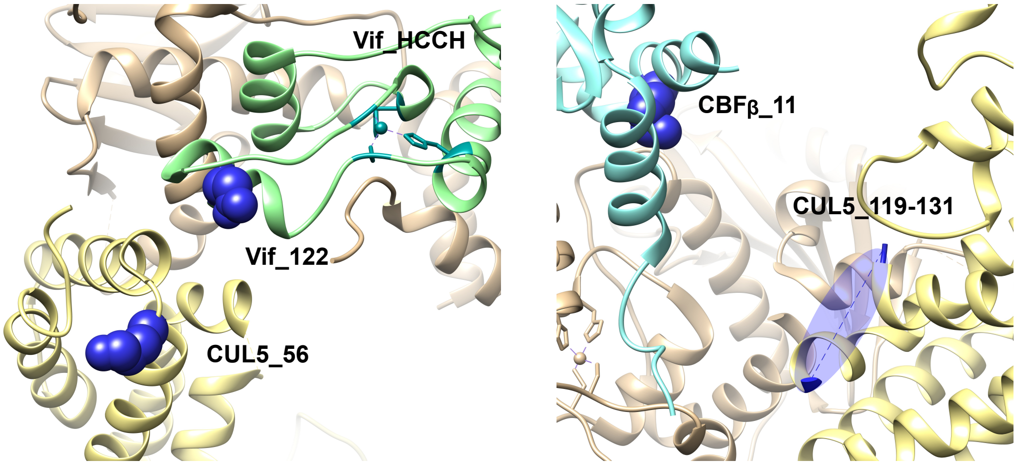


**Figure S6. Structural features of Vif-CBC-Cul5$_{NTD}$ captured by cross-linking mass spectrometry. A.** Zoomed in view of the X-ray structure (PDB: 4N9F) showing cross-linked residues of Vif K122 and Cul5 K56. Vif is colored green and Cul5 yellow. Cross-linked residues are shown in blue. The Vif HCCH domain and coordinating zinc ion are shown in blue-green. **B.** Zoomed in view of the X-ray structure showing cross-linked residues CBFβ K11 and Cul5 K126 and K127 (missing segment Cul5 119-131 is shown as a blue ellipse). CBFβ is colored light blue and Cul5 yellow. Cross-linked residues are shown in blue.

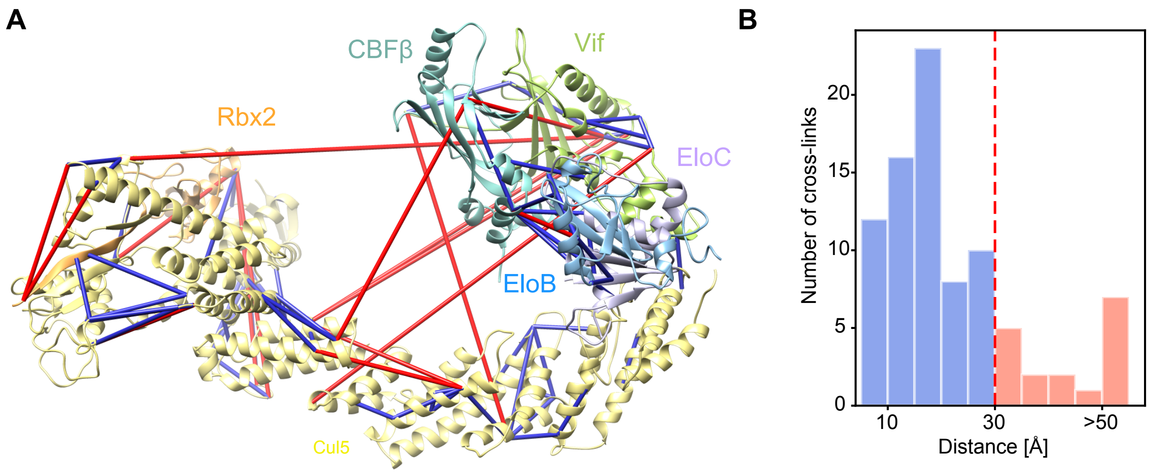


**Figure S7. DSSO cross-links mapped to the comparative model of Vif-CRL5-CBFβ. A.** The Vif-CRL5-CBFβ comparative model was built based on the atomic structure of the Vif-CBC-Cul5$_{NTD}$ subcomplex (PDB:49NF) and comparative models of the Cul5 CTD and Rbx2 (Supporting information). Shown in blue are cross-links that satisfy expected Cα-Cα distances (< 30 Å) based on the linker length, lysine side chain length, and flexibility of the protein backbone. Shown in red are cross-links that violate the expected Cα-Cα distance. **B.** Histogram showing the distribution of the cross-linked Cα–Cα distances in the Vif-CRL5-CBFβ comparative model. 87 out of 132 unique linkages map to the Vif-CRL5-CBFβ comparative structure, with 80% being satisfied.

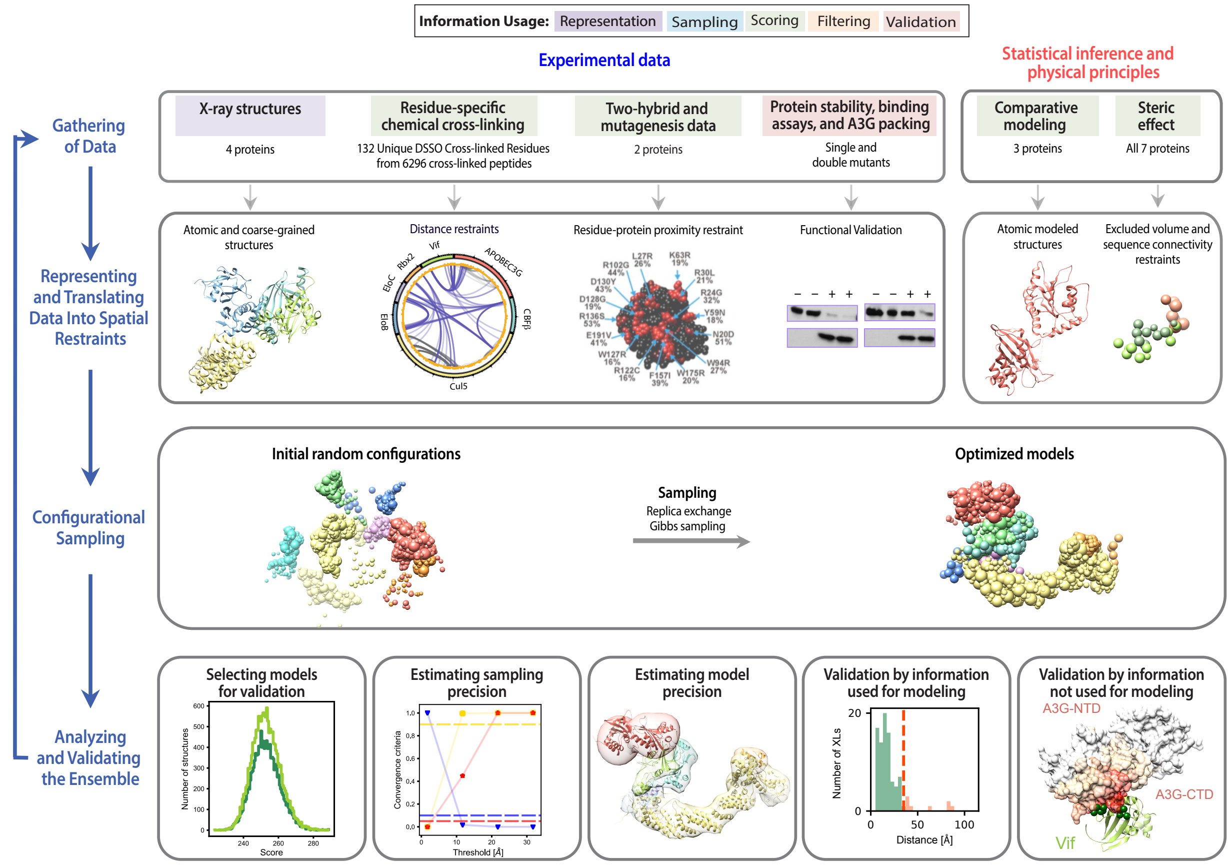


**Figure S8. The four-stage scheme for integrative structure modeling of the A3G-Vif-CRL5-CBFβ complex.** Our integrative approach proceeds through four stages: (1) gathering of data, (2) representation of subunits and translation of the data into spatial restraints, (3) configurational sampling to produce an ensemble of models that satisfies the restraints, and (4) analysis and validation of the ensemble.

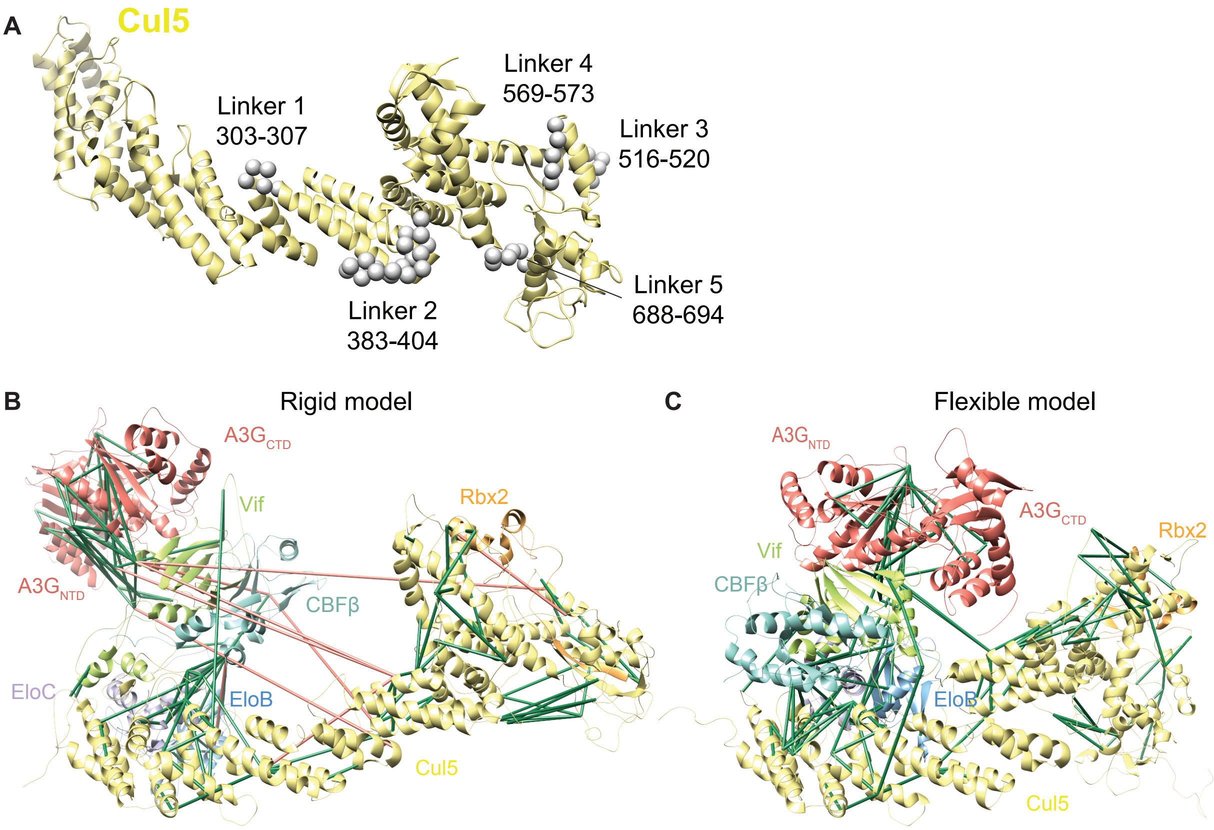


**Figure S9. Integrative structure modeling of the A3G-Vif-CRL5-CBFβ complex. A.** Comparative model of the full-length Cul5 showing the domain organization. Cul5 was represented as five rigid bodies connected by flexible linkers (grey spheres). **B.** Detail of crosslinks mapped to the centroid structure of the rigid model of the A3G-Vif-CRL5-CBFβ complex. Satisfied and violated crosslinks shown in green and pink, respectively. **C.** Detail of crosslinks mapped to the centroid structure of the flexible model of the A3G-Vif-CRL5-CBFβ complex.

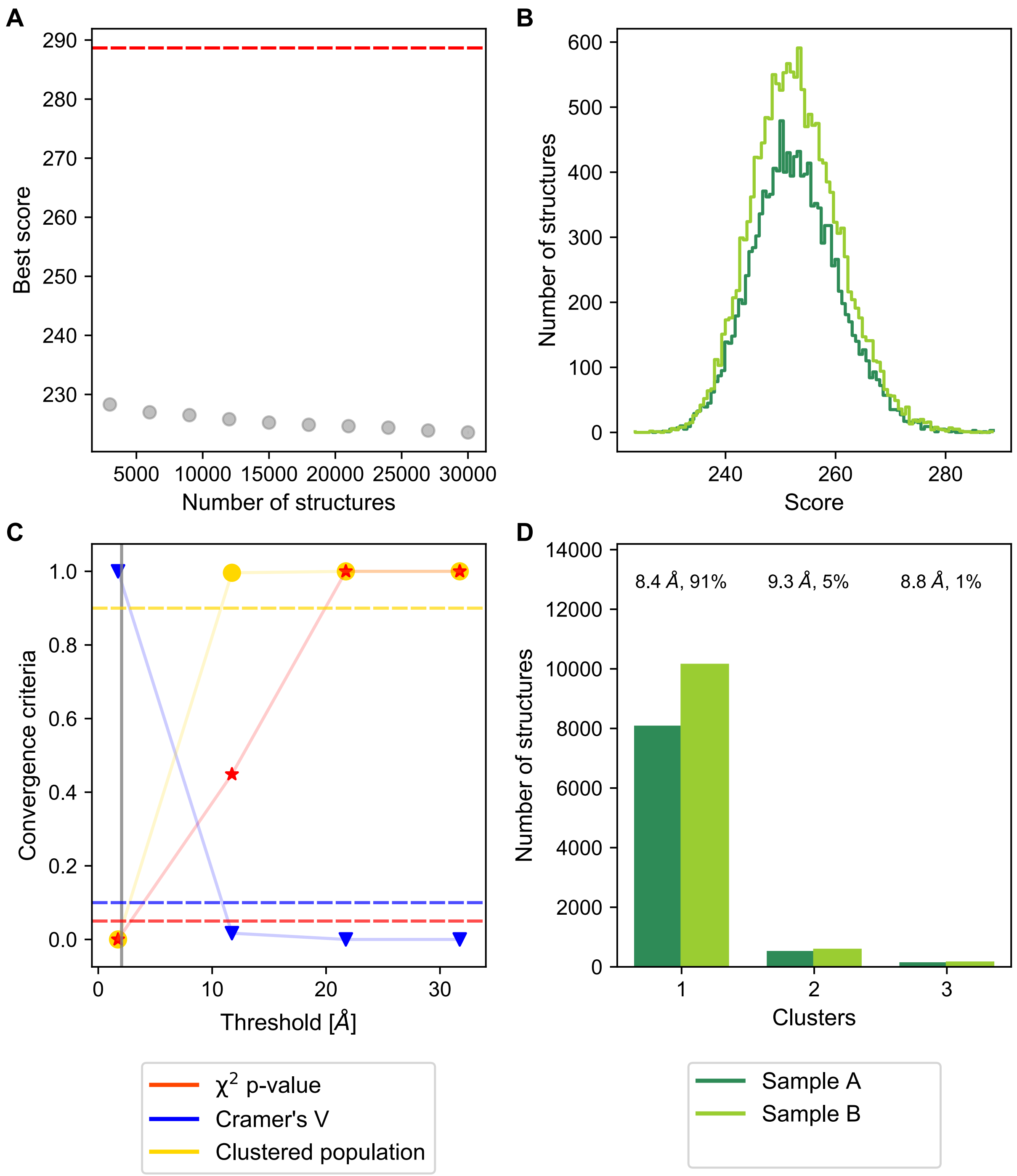


**Figure S10. Estimation of sampling precision for A3G-Vif-CRL5-CBFβ using the rigid representation. A.** Convergence of the model scores in the ensemble. Grey dots show that the scores do not continue to improve as more structures are independently computed. The dotted line indicates the highest score in the ensemble. **B.** Distribution of scores for structures in samples A (dark green) and B (light green), comprising 20,000 random models in the ensemble. The non-parametric Kolmogorov-Smirnov two-sample test (two sided) indicates that the difference between the two score distributions is insignificant (p-value (0.18)>0.05). In addition, the

magnitude of the difference is small, as demonstrated by the Kolmogorov-Smirnov two-sample test statistic (D=0.57). **C.** Three criteria for determining the sampling precision (y-axis), evaluated as a function of the RMSD clustering threshold (x-axis). First, the p-value is computed using the $\chi2$-test (one-sided) for homogeneity of proportions (red stars). Second, an effect size for the $\chi2$-test is quantified by the Cramer's V value (blue triangles). Third, the population of structures in sufficiently large clusters (containing at least ten structures from each sample) is shown as yellow circles. The vertical dotted grey line indicates the RMSD clustering threshold at which three conditions are satisfied ($\chi2$-test p-value (0.449) > 0.05 (red, horizontal dotted line), Cramer's V (0.017) < 0.10 (blue, horizontal dotted line), and the population of clustered structures (0.967) > 0.80 (yellow, horizontal dotted line)), thus defining the sampling precision of 11.7 Å. The three solid curves (in red, blue, and yellow) were drawn through the points to help visualize the results. **D.** Population of structures in samples A and B in each of the three clusters obtained by threshold-based clustering using an RMSD threshold of 11.7 Å. The dominant cluster (cluster 1) contains 92% of the structures. Cluster precision and population is shown for each cluster. The precision of the dominant cluster defines the model precision.

**A**

| Simulation | Rigid model | Flexible model | No mutagenesis data | Only mutagenesis data | Jackknifing with random draw of 80% of cross-links | Jackknifing with random draw of 80% of cross-links | Jackknifing with random draw of 60% of cross-links | Jackknifing with random draw of 60% of cross-links | No cross-links composite confidence score |
|---|---|---|---|---|---|---|---|---|---|
| **Sampling precision [Å]** | 11.7 | 29 | 20 | 19.7 | 11.6 | 11.6 | 15.6 | 22 | 16 |
| **Number of clusters** | 2 | 1 | 1 | 2 | 1 | 1 | 1 | 1 | 2 |
| **Model precision [Å]** | 8.4 | 19.9 | 13.7 | 13.7,9.9 | 7.7 | 7.8 | 9.9 | 14.4 | 10.5, 9.1 |
| **Clusters population (%)** | 92, 5 | 98.7 | 72 | 67,7 | 73 | 73 | 71 | 75 | 50,25 |
| **XLs satisfaction (used for modeling, %)** | 89 | 99 | 89 | NA | 90 | 90 | 87 | 91 | 89 |
| **XLs satisfaction (not used for modeling, %)** | NA | NA | NA | 87 | 84 | 88 | 94 | 88 | NA |
| **Genetics satisfaction (used for modeling, %)** | 98 | 98 | NA | 93 | 96 | 96 | 93 | 94 | 94 |
| **Genetics satisfaction (not used for modeling, %)** | NA | NA | 10 | NA | NA | NA | NA | NA | NA |

**B**

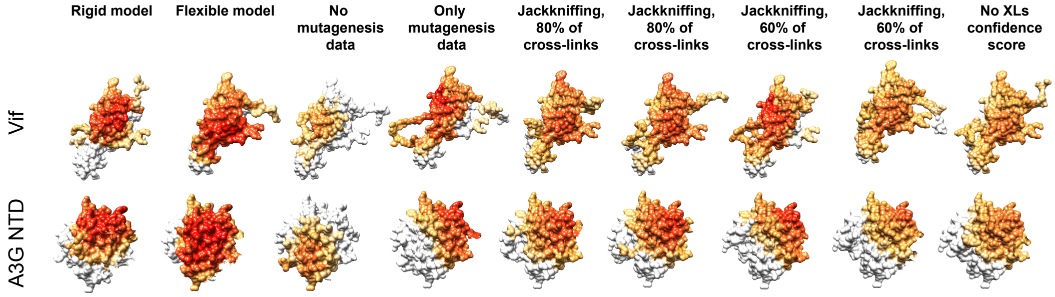


**Figure S11. Validation of integrative models. A.** To quantify the degree to which a model satisfied relevant information not used to compute it, we recomputed the A3G-Vif-CRL5-CBFβ integrative structure excluding parts of the input information. In particular, we recomputed the models omitting the restraints derived from mutagenesis studies, the cross-links data or a fraction of it (i.e., jackknifing), or including all the data but omitting the composite confidence scores derived for each cross-link. In all cases, we report the sampling and model precision, and we compared the models against the omitted data, to validate both the model and the cross-links. **B.** Surface representation of Vif (top) and A3G (bottom) showing the binding interface with the intensity of red proportional to the fraction of models in the main cluster whose distance is closer than the cutoff of 12 Å.

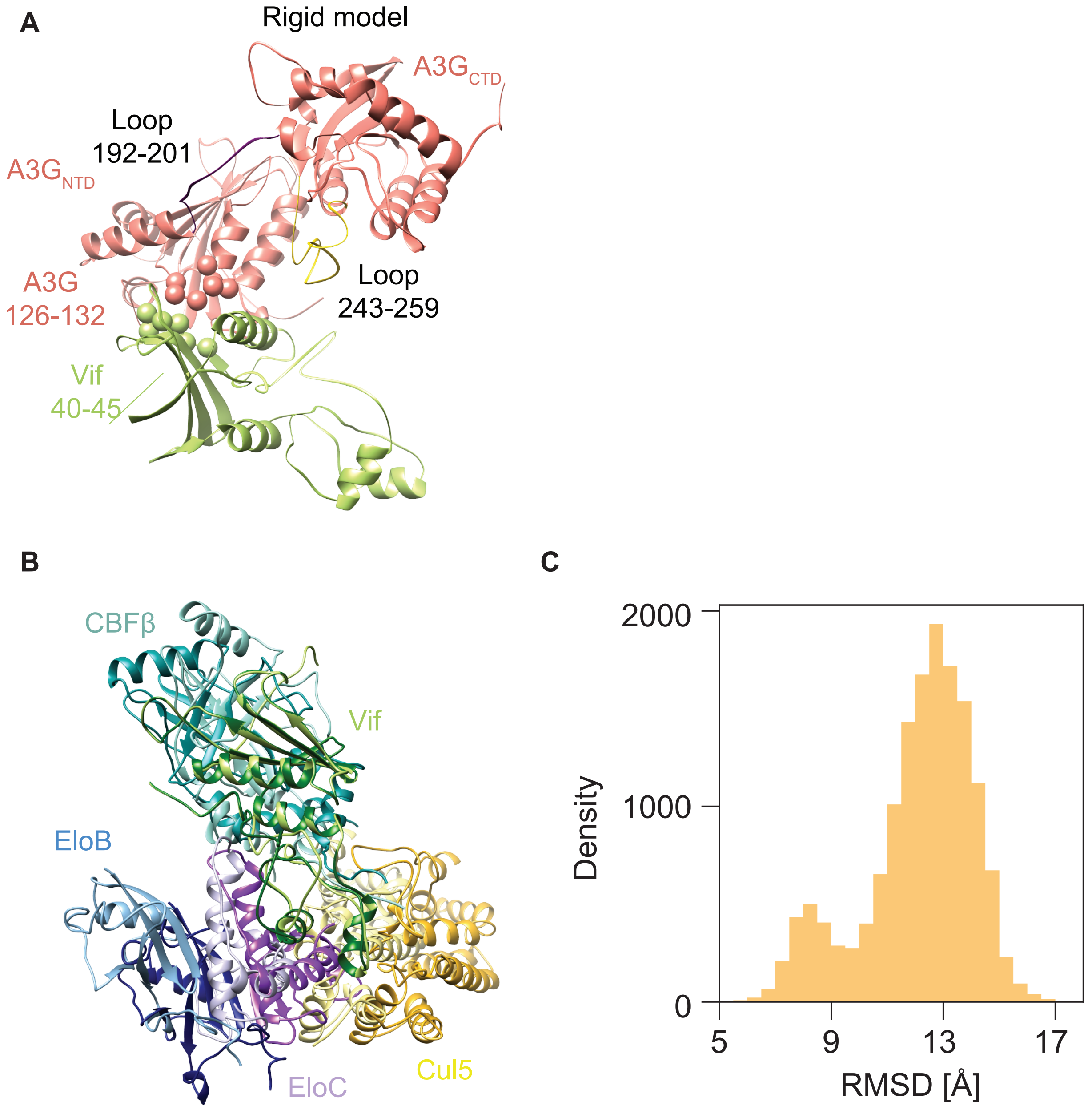


**Figure S12. Flexibility of the A3G-Vif-CRL5-CBFβ complex. A.** A3G domain organization. The A3G NTD and CTD and shown with loop 192-200 (dark red) connecting the two domains. The A3G CTD loop 243-259 is shown in yellow. Segments A3G 126-132 and Vif 40-45 are shown as spheres. **B.** Comparison of the Vif-CBC-Cul5$_{NTD}$ subcomplex in the rigid model (i.e., the X-ray structure) and the centroid of the flexible model. The subunits of the flexible model are shown in dark colors. **C.** Distribution of RMSD between the rigid model (i.e., the X-ray structure) and all the computed structures in the flexible model ensemble after superimposing the C$\alpha$ coordinates of the Vif-CBC-Cul5$_{NTD}$ subcomplex.

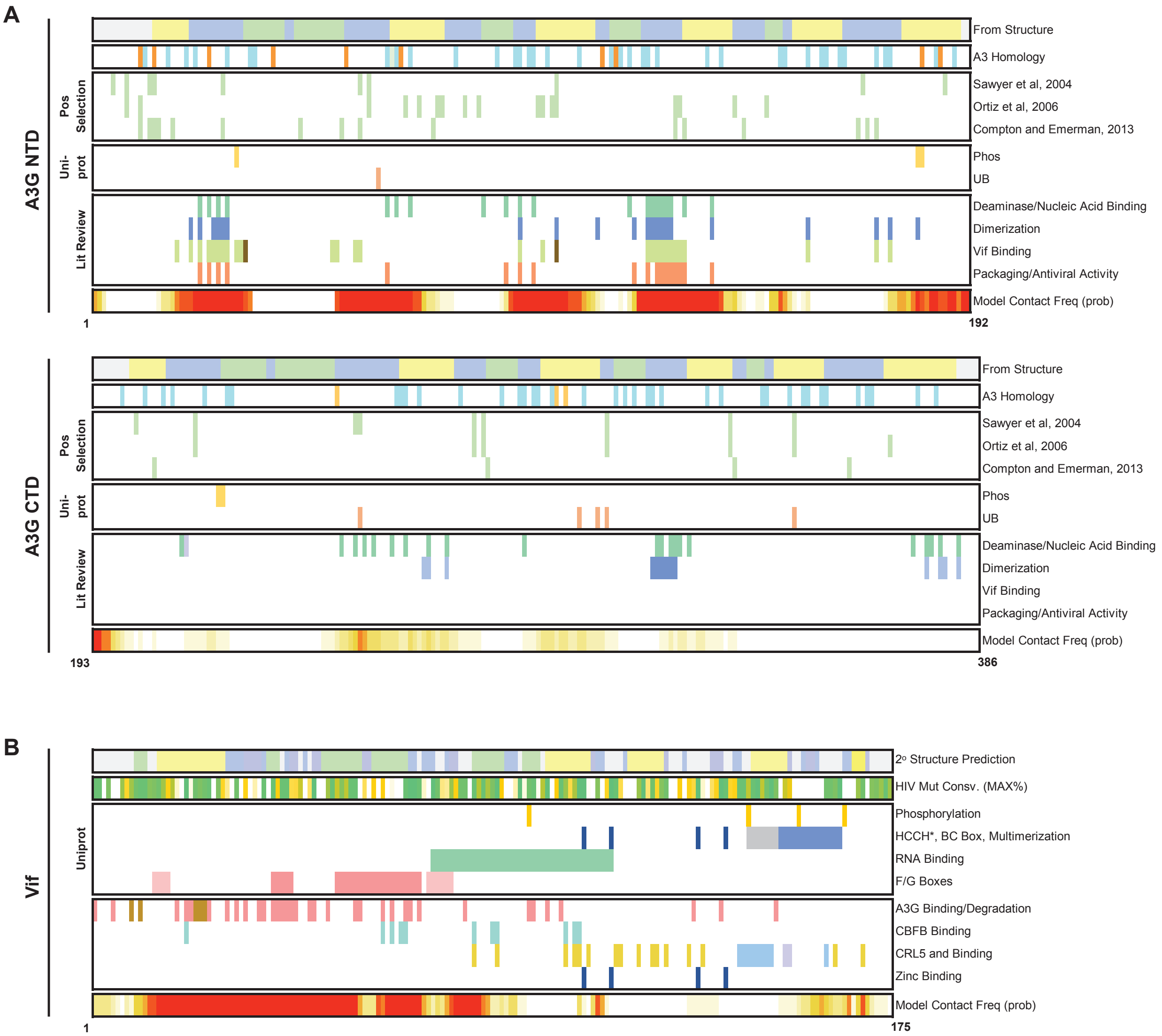


**Figure S13.** Graphical representation of **Table S9** and **S10** demonstrating A3G-Vif predicted interface, and previously known structural and functional features for Vif and A3G.

**Supplemental Table S1.** Metadata summary of all sample preparation and data processing for each MS run in the full XL-MS dataset.

**Supplemental Table S2.** Unique inter-linked peptides used in all integrative models for A3G-Vif-CRL5-CBFβ structure determination.

**Supplemental Table S3.** Full list of all DSSO inter-linked peptides with corresponding detailed Protein Prospector scores and information.

**Supplemental Table S4.** Full list of all DSSO loop-linked peptides with corresponding detailed Protein Prospector scores and information.

**Supplemental Table S5.** Full list of all DSSO dead-end (mono-linked) peptides with corresponding detailed Protein Prospector scores and information.

**Supplemental Table S6.** Redundant counts of all DSSO cross-links (inter-subunit, intra-subunit, and dead-end) by subunit and linkable residue (Lys or N-terminal). Intra-subunit linkages are the sum of inter-linked and loop-linked data.

**Supplemental Table S7.** Summary of the integrative structure determination, thoroughness of configurational sampling, structure precision, and validation of A3G-Vif-CRL5-CBFβ (rigid representation).

**Supplemental Table S8.** Summary of the integrative structure determination, thoroughness of configurational sampling, structure precision, and validation of A3G-Vif-CRL5-CBFβ (flexible representation).

**Supplemental Table S9.** Summary of A3G sequence, structure, evolution, and function information by residue based on(21, 34, 40, 42, 44, 75, 85–88, 93, 100, 136, 140–148).

**Supplemental Table S10.** Summary of Vif sequence, structure, mutation frequency (HIVMut DB), and function information by residue based on(32, 41, 42, 44, 50, 75, 88, 93, 100, 101, 118, 130, 145, 147, 149–177).

**Supplemental File bt_typsin.xml.** Bash scripts were used to automate the job submissions and processing of hundreds of files. This is the Parameters file for ProteinProspector Batchtag searches for all trypsin digested samples, where "xxx" indicates individual filenames from each of the different MS3.mgf extracted files.

**Supplemental File bt_chymotpsin.xml.** Bash scripts were used to automate the job submissions and processing of hundreds of files. This is the Parameters file for ProteinProspector Batchtag searches for all chymotrypsin digested samples, where "xxx" indicates individual filenames from each of the different MS3.mgf extracted files.

**Supplemental File sc.xml.** Bash scripts were used to automate the job submissions and processing of hundreds of files. This is the Parameters file for ProteinProspector SearchCompare for all trypsin and chymotrypsin digested samples, where "xxx" indicates individual filenames from each of the different MS3.mgf extracted files.

**Supplemental File Usermod_xlink.txt.** In order to complete the searches in ProteinProspector, additional modifications needed to be added to the local usermod_xlink.txt file in the Protein Prospector package. The XL:A modifications for uncleaved Lysines and Protein-N termini were added as indicated.
