# Supplementary material for: Characterization of an A3G-VifHIV-1-CRL5-CBFβ Structure Using a Cross-linking Mass Spectrometry Pipeline for Integrative Modeling of Host–Pathogen Complexes
Source: Mol Cell Proteomics. 2021 Aug 11;20:100132. doi: 10.1016/j.mcpro.2021.100132 (PMC8459920; doi:10.1016/j.mcpro.2021.100132)
Supplement: Supplemental Table S8 [file mmc15.pdf]

Table 8: Summary of Integrative Structure Determination of A3G_Vif_CRL5 with flexible representation

**1) Gathering information**	
Prior models	CBFB: comparative model, template 4N9F:F<br>Vif: comparative model, template 4N9F:G<br>EloB: comparative model, template 4N9F:D<br>EloC: comparative model, template 4N9F:E<br>CUL5: comparative model, template 4N9F:C<br>Rbx2: comparative model, template 1LDJ:B<br>A3G: comparative model, template 5K81:E
Physical principles and statistical preferences	Excluded volume<br>Sequence connectivity
Experimental data	131 DSSO chemical cross-links<br>Predicted residue-protein contacts from mutagenesis studies; A3G residues 126-132 and Vif residues 40-45
**2) Representing the system**	
Composition (number of copies)	A3G: 1<br>CBFB: 1<br>CUL5: 1<br>EloB: 1<br>EloC: 1<br>Rbx2: 1<br>Vif: 1
Atomic (structured) components	A3G: 6-194, 200-243, 258-380<br>CBFB: 1-156<br>CUL5: 11-302, 308-382, 405-515, 521-568, 574-687, 695-780<br>EloB: 1-105<br>EloC: 17-112<br>Rbx2: 27-113<br>Vif: 6-154, 166-175
Unstructured components	A3G: 1-5, 195-199, 244-257, 381-384<br>CBFB: 157-182<br>CUL5: 1-10, 303-307, 383-404, 516-520, 569-573, 688-694<br>EloB: 106-161<br>EloC: 1-16<br>Rbx2: 1-26<br>Vif: 1-5, 155-165
Resolution of structured components	1 [R1], 10 [R10] residues per bead
Resolution of unstructured components	5 [R5] residues per bead
Structural coverage	89.02 %
Rigid body (RB) definitions	RB1: $CBFB_{1-156}$<br>RB2: Vif_{6-154},$Vif_{166-175}$<br>RB3: $EloB_{1-105}$<br>RB4: $EloC_{17-112}$<br>RB5: $CUL5_{11-302}$<br>RB6: $CUL5_{308-382}$<br>RB7: $CUL5_{405-515}$<br>RB8: $CUL5_{521-568}$<br>RB9: $CUL5_{574-687}$<br>RB10: $CUL5_{695-780}$<br>RB11: $Rbx2_{27-113}$<br>RB12: $A3G_{6-194}$<br>RB13: $A3G_{200-243}$,$A3G_{258-380}$
Spatial restraints encoded into scoring function	Excluded volume; applied to the R1 representation<br>Sequence connectivity; applied to the R1 representation<br>Cross-link restraints; applied to the R1 representation<br>Residue-protein proximity restraints; applied to the R1 representation<br>Structural equivalence distance restraints; applied to the R1 representation
**3) Structural Sampling**	
Sampling method	Replica Exchange Gibbs sampling, based on Metropolis Monte Carlo
Replica exchange temperature range	1.0 - 2.5
Number of replicas	8

Number of runs	50
Number of structures generated	3000000
Movers for flexible string of bead	Random translation up to 4.0 Å
CPU time	6 hours on 20 processors
**4) Validating the model**	
**Models selected for validation**	
Number of models after equilibration	3000000
Number of models that satisfy the input information	203100
Number of structures in samples A/B	102925/100175
p-value of non-parametric Kolmogorov-Smirnov two-sample test	0.05 (threshold p-value > 0.05)
Kolmogorov-Smirnov two-sample test statistic, D	1.0
**Thoroughness of the structural sampling**	
Sampling precision	28.98 Å
Homogeneity of proportions χ^2 test (p-value)/Cramers V value	0.000/0.043 (thresholds: p-value>0.05 OR Cramer's V<0.1)
Number of clusters	1
Cluster populations	cluster 1 : 98.7 %
Cluster precisions	cluster 1 : 19.85 Å
Average cross-correlation between localization probability densities of samples A and B	cluster 1: 0.9
**Validation by information used for modeling**	
Percent of sequence connectivity restraints satisfied per structure	99 %
Percent cross-link restraints satisfied by ensemble	99 %
Percent of residue-protein proximity restraints satisfied by ensemble	98 %
Percent of excluded volume restraints satisfied per structure	97 %
**5) Benchmark**	
**6) Software and data availability**	
**Software**	
Modeling programs	IMP PMI module, version develop-af393bce43<br>Integrative Modeling Platform (IMP), version develop-af393bce43<br>MODELLER, version 9.20<br>MODELLER, version 9.19
Modeling scripts	https://github.com/integrativemodeling/A3G_Vif_CRL5
Homology detection and structure prediction	HHPred, version 2.0.16
Visualization and plotting	UCSF Chimera, version 1.10<br>Matplotlib, version 3.0.3
**Data**	
PDB-dev accesion code	TBD
